# Atypical genotoxicity of carcinogenic nickel(II): Linkage to dNTP biosynthesis, DNA-incorporated rNMPs, and impaired repair of TOP1-DNA crosslinks

**DOI:** 10.1016/j.jbc.2023.105385

**Published:** 2023-10-25

**Authors:** Casey Krawic, Michal W. Luczak, Sophia Valiente, Anatoly Zhitkovich

**Affiliations:** Department of Pathology and Laboratory Medicine, Brown University, Providence, Rhode Island, USA

**Keywords:** ribonucleotide reductase, nickel, replication stress, DNA-embedded ribonucleotides, DNA-topoisomerase I crosslink

## Abstract

Cancer is a genetic disease requiring multiple mutations for its development. However, many carcinogens are DNA-unreactive and nonmutagenic and consequently described as nongenotoxic. One of such carcinogens is nickel, a global environmental pollutant abundantly emitted by burning of coal. We investigated activation of DNA damage responses by Ni and identified this metal as a replication stressor. Genotoxic stress markers indicated the accumulation of ssDNA and stalled replication forks, and Ni-treated cells were dependent on ATR for suppression of DNA damage and long-term survival. Replication stress by Ni resulted from destabilization of RRM1 and RRM2 subunits of ribonucleotide reductase and the resulting deficiency in dNTPs. Ni also increased DNA incorporation of rNMPs (detected by a specific fluorescent assay) and strongly enhanced their genotoxicity as a result of repressed repair of TOP1-DNA protein crosslinks (TOP1-DPC). The DPC-trap assay found severely impaired SUMOylation and K48-polyubiquitination of DNA-crosslinked TOP1 due to downregulation of specific enzymes. Our findings identified Ni as the human carcinogen inducing genome instability *via* DNA-embedded ribonucleotides and accumulation of TOP1-DPC which are carcinogenic abnormalities with poor detectability by the standard mutagenicity tests. The discovered mechanisms for Ni could also play a role in genotoxicity of other protein-reactive carcinogens.

Malignant transformation of cells results from accumulation of oncogenic mutations caused by DNA damage and replication errors (1). Recognition of cancer as a genetic disease has led to testing of consumer products for their ability to cause DNA damage and mutations to prevent human exposure to potential carcinogenic hazards. However, many complete human carcinogens (including their metabolites) are not DNA-reactive and do not test positive for mutagenicity (2, 3, 4, 5). A common interpretation of the negative mutagenicity results is that these carcinogens must be acting *via* nonmutagenic mechanisms, which is true for some but not necessarily all chemicals in this group. The existence of apparently nonmutagenic carcinogens also raises a question whether some forms of oncogenic DNA abnormalities are poorly detectable by the standard mutagenicity tests such as the Ames bacterial reverse mutation assay and the *Hprt* or *Tk* forward mutation assays in mammalian cells. Both Ames and mammalian tests are responsive to carcinogens generating alkylation and oxidative DNA damage, with the latter frequently being suspected as an indirect source of genotoxic lesions by DNA-unreactive carcinogens.

It has recently become evident that DNA-embedded ribonucleotides (rNMPs) represent another significant endogenous form of abnormalities in the human genome (6, 7, 8). Overabundance of rNMPs in nuclear DNA due to their defective repair caused by mutations in RNase H2 induces genomic instability (9, 10, 11, 12, 13), replication stress (11, 14), and activation of the DNA damage response and p53 (9, 10, 15). Mutagenicity of DNA-embedded rNMPs has been largely studied in yeast. Deletion of the catalytic subunit of RNaseH2 in yeast was found to moderately increase the number of mutants at two loci and produce no mutants at the third locus (16). Subsequent studies determined that rNMP-induced mutagenesis was strongly sequence-dependent and primarily involved generation of small 2- to 5-bp deletions (17, 18), arising as a result of mutagenic processing of ribonucleotides in DNA by topoisomerase I in the areas of short repeats (19, 20). In yeast, topoisomerase I-mediated cleavage at DNA-embedded rNMPs can also produce DNA double-strand breaks (21). The carcinogenic role of DNA-incorporated rNMPs was demonstrated by the formation of tumors in mice with the tissue-specific loss of functional RNase H2 such as squamous cell carcinoma in the skin with disrupted RNase H2 in epidermal cells (22). Deletion of RNase H2 in mouse intestinal epithelial tissue induced the development of intestinal and colorectal carcinomas (23). It has recently been determined (24) that DNA-incorporated rNMPs processed by topoisomerase I were also the cause of the ID4 pancancer mutation signature characterized by short 2 to 5 bp deletions (25). Despite clear evidence of their genotoxic activity and carcinogenic potential, DNA-incorporated rNMPs have not yet been linked to genome instability by any known human carcinogen.

Nickel (Ni) is a global environmental pollutant and a firmly established human carcinogen (26, 27, 28, 29, 30). The predominant malignancy caused by Ni exposures is squamous lung carcinoma (31), a tumor type containing the highest mutational load among 12 main human internal cancers (32). Ni is a major industrial metal used in plating and production of stainless steel (the largest-volume metal alloy in the world), coins, jewelry, fuel cells, and Ni-Cd batteries. Environmental release of Ni and its compounds annually in the United States is estimated at approximately 40 million pounds (26). Globally, the main source of atmospheric Ni is burning of coal (33), which annually releases >3 million tons of Ni in China alone (34). Atmospheric emissions of Ni grow at ∼5% annual rate. Carcinogenicity of various Ni compounds is caused by Ni(II) ions delivered directly into cells or released intracellularly from internalized particles (35, 36, 37). However, further steps in Ni carcinogenicity are more poorly understood. Ni(II) is unreactive toward DNA and Ni(II) compounds were not mutagenic in the standard tests such as *Salmonella* reverse mutagenesis (38), rodent *Hprt* (39) and ouabain forward mutation assays (40). Consequently, Ni is typically described as a nongenotoxic carcinogen, and it is even used as a negative control in the development of high-throughput screens for genotoxicants.

In this work, we investigated upstream mechanisms of p53 activation by Ni(II) in human lung cells and unexpectedly found that this metal was a replication stressor destabilizing RRM1 and RRM2 subunits of the dNTP-producing ribonucleotide reductase. Insufficient supply of dNTPs was associated with increased DNA incorporation of rNMPs whose genotoxicity was strongly increased by Ni(II) due to its repressive effects on the SUMO-dependent repair of TOP1-DNA-protein crosslinks (TOP1-DPC). Our findings identified Ni(II) as the first human carcinogen causing genotoxicity through DNA-embedded ribonucleotides and accumulation of TOP1-DPC. These mechanisms can potentially be involved in atypical genotoxicity of other protein-reactive carcinogens with weak or no responses in the standard mutagenicity tests. Our new assays for detection of DNA-incorporated rNMPs and TOP1-DPC provide sensitive methodologies for screening of other toxicants for their ability to increase DNA burden of ribonucleotides and DNA-trapped TOP1.

## Results

### Activation of DNA damage responses by Ni(II)

**Figure 1:**
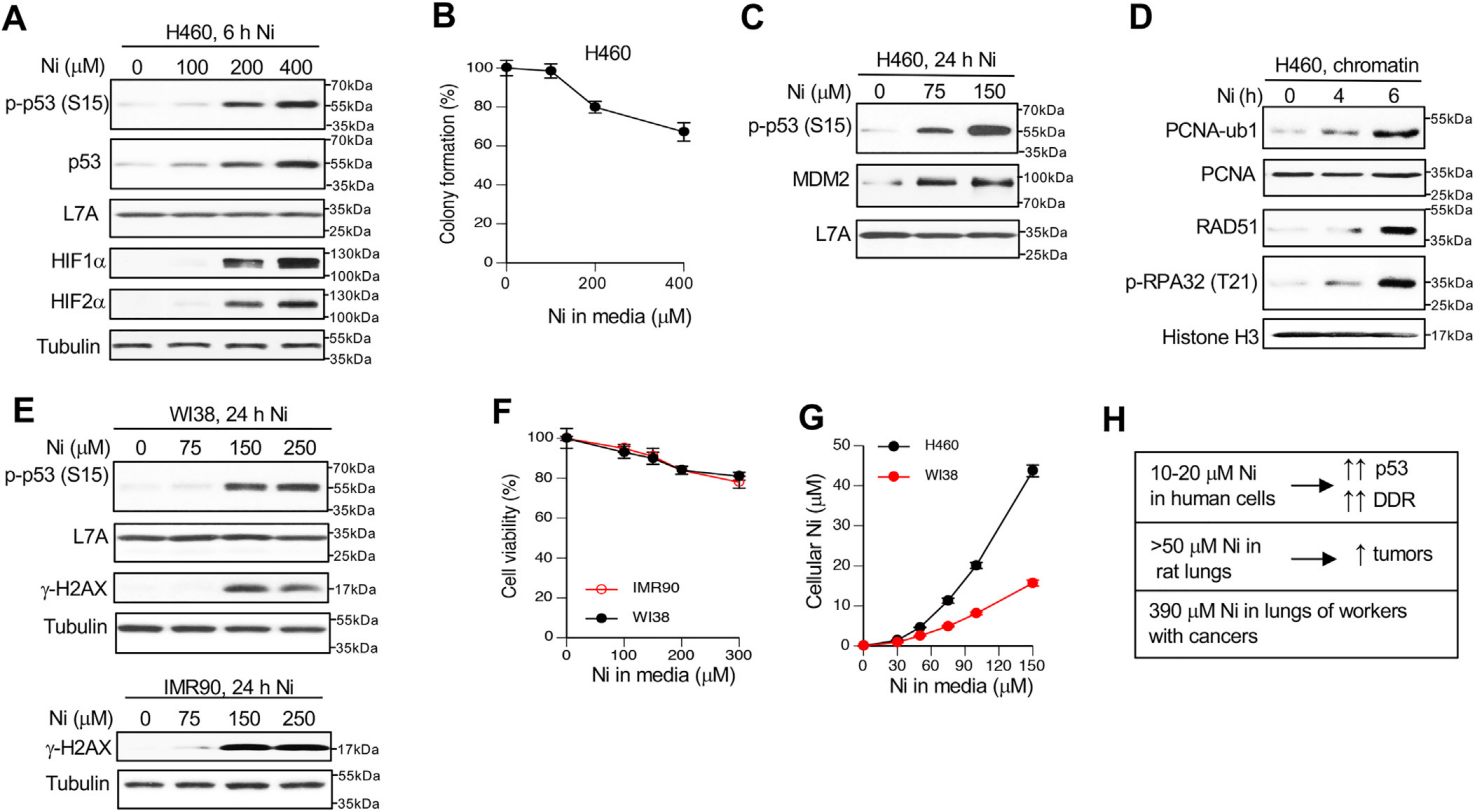
**Activation of DNA damage responses by Ni(II) in human cells.** Whole-cell lysates were used for immunoblotting in all panels except *panel D*. *A*, activation of p53 and hypoxia response pathways in H460 cells treated with Ni(II) for 6 h. *B*, colony formation by H460 cells treated with Ni(II) for 6 h. Data are means ± SD, n = 3. *C*, MDM2 levels and p53-S15 phosphorylation in H460 treated with Ni(II) for 24 h. *D*, immunoblots of chromatin-bound proteins in H460 cells treated with 400 μM Ni(II) for 0, 4 or 6 h (PCNA-ub1: a monoubiquitinated form detected in overexposed PCNA blots). *E*, histone H2AX-S139 (γ-H2AX) and p53-S15 phosphorylation in primary human cells treated with Ni(II) for 24 h. *F*, viability of IMR90 and WI38 primary human cells treated with Ni(II) for 24 h and assayed 48 h later. Data are means ± SD, n = 3. *G*, intracellular concentrations of Ni(II) in H460 and WI38 cells treated for 24 h (means ± SD, n = 3). *H*, summary of the results.

As discussed in the Introduction, Ni(II) does not react with DNA and is not mutagenic in the standard assays. However, it causes chromosomal abnormalities (41, 42, 43) that have been attributed to damaged microtubules but could also result from DNA damage. A common response to DNA damage is activation of the transcription factor p53 which is a target of multiple stress signaling pathways (44, 45). Upregulation of p53 was important for apoptotic death and clonogenic lethality by Ni(II) (46) although the nature of Ni(II)-induced injury triggering this response remained unknown. We reasoned that a potentially informative approach for the exploration of potential genotoxic properties of Ni could be the investigation of DNA damage responses, especially the identification of upstream activators of p53. We used H460 human lung epithelial cell line as our primary model which has wild-type p53 and showed normal DNA damage responses to ionizing radiation (47) and chemical genotoxicants (48, 49). We found that short-term treatments of H460 cells with Ni(II) produced a clear upregulation of p53, as evidenced by strong increases in its S15 phosphorylation and protein accumulation (Fig. 1*A*). The dose dependence of p53 activation followed very closely the upregulation of the hypoxia-inducible factors HIF1α and HIF2α, which is a known nongenotoxic response to the accumulation of cellular Ni(II) (35, 37). The doses of Ni causing p53 activation in short-term treatments were only moderately cytotoxic as measured by the colony formation assay (Fig. 1*B*). In longer 24-h treatments, p53 phosphorylation and upregulation of the p53 transactivation target MDM2 required even lower doses of Ni(II) (Fig. 1*C*). To explore other components of the DNA damage response, we examined chromatin from H460 cells treated with Ni(II) for 4 and 6 h, which corresponds to the initial wave of p53 activation. We found that chromatin of Ni(II)-treated cells contained elevated amounts of monoubiquitinated PCNA (Fig. 1*D*) which is a marker of stalled replicative DNA polymerases (50). Ni also stimulated a higher chromatin binding of the central homologous recombination protein RAD51 and induced RPA32-T21 phosphorylation which occurs in cells with damaged replication forks (51). To exclude a possibility that transformed H460 cells were somehow unique in their ability to activate DNA damage responses by Ni, we next examined two primary human lung cell lines: WI38 and IMR90. Ni(II) treatments of primary human cells also induced a robust phosphorylation of p53 at S15 and histone H2AX at S139 (γ-H2AX) (Fig. 1*E*). γ-H2AX is a marker of severe genotoxic stress that is most commonly linked to the production of DNA double-stranded breaks (52). Ni(II) doses that strongly increased p53 and H2AX phosphorylation in primary cells were only minimally cytotoxic (Fig. 1*F*). Cultured cells poorly accumulate Ni(II) ions due to their binding by media components and a robust efflux. To determine a more exact relationship between external and internal concentrations of Ni(II), we measured its cellular concentrations by atomic absorption spectroscopy. We found that doses triggering activation of p53 and other DNA damage responses delivered ∼10 to 15 μM cellular Ni (Fig. 1*G*). The dose dependence of external *versus* internal concentrations was sublinear for both H460 and WI38 cells, with cellular levels representing only 3 to 10% of those in media of primary cells. Tissue metal measurements in animal carcinogenicity studies showed that tumors appeared at >50 μM Ni in the rat lung (53). Ni-exposed workers with lung cancer had on average 390 μM Ni in the adjacent healthy tissue (54). Thus, the DNA damage markers in our studies were strongly induced at cellular Ni levels that are relevant to lung carcinogenicity (summarized in Fig. 1*H*).

### Importance of ATR kinase for Ni(II) tolerance

**Figure 2:**
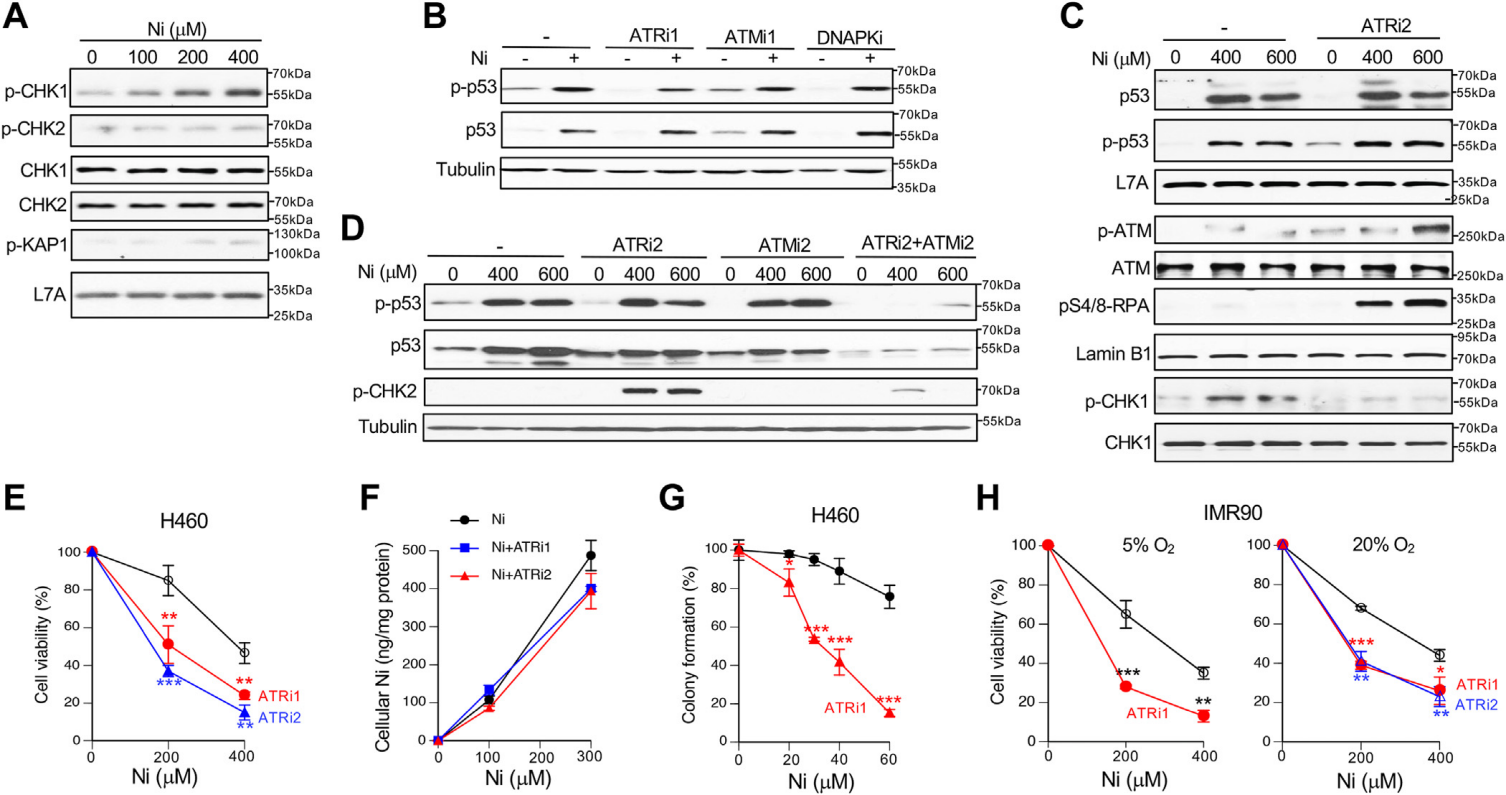
**Importance of ATR kinase in p53 activation and cell resistance to Ni(II).** H460 cells were treated with Ni(II) for 6 h in the presence of various inhibitors to identify a p53-targeting kinase: ATRi1: 1 μM AZD6738, ATRi2: 10 μM VE821, ATMi1: 10 μM KU55933, ATMi2: 1 μM KU60019, DNAPKi: 30 μM NU7026. *A*, phosphorylation levels of CHK1 (S317), CHK2 (T68) and KAP1 (S824) in Ni(II)-treated H460 cells. *B*, S15-p53 phosphorylation and p53 protein levels in the presence of inhibitors of DNA damage-responsive kinases (ATR, ATM, DNAPK). *C*, effects of inhibition of ATR alone and (*D*) ATR and ATM kinases together on p53 upregulation and phosphorylation of ATM targets (ATM-S1981, CHK2-T68). *E*, viability of H460 cells treated with Ni(II) for 18 h and assayed 48 h later (ATRi1: 0.5 μM AZD6738, ATRi2: 10 μM VE821). Means ± SD, n = 3, ∗∗*p* < 0.01, ∗∗∗*p* < 0.001. *F*, Ni(II) accumulation in H460 cells in the absence and presence ATR inhibitors. Cells were treated as in panel E and collected immediately for metal measurements. Data are means ± SD, n = 3. *G*, colony formation by Ni(II)-treated H460 cells in the absence and presence of 0.3 μM AZD6738 (ATRi1). Means ± SD, n = 3, ∗*p* < 0.05, ∗∗*p* < 0.01, ∗∗∗*p* < 0.001. *H*, viability of IMR90 cells grown and treated with Ni(II) under 5 or 20% O_2_. Cells were treated with Ni(II) for 48 h and assayed 24 h later. ATRi1: 0.3 μM AZD6738, ATRi2: 5 μM VE821. Data are means ± SD, n = 3, ∗∗*p* < 0.01, ∗∗∗*p* < 0.001.

Signaling from multiple stress-activated pathways converges on the transcriptional factor p53 which controls major cellular responses such as cell cycle checkpoints, DNA repair, cell metabolism, and cell fate decisions. Stress signal transduction is initiated by apical stress-specific kinases that phosphorylate p53 as their major downstream target (44, 45). Thus, identification of the p53-targeting kinase can help characterize the nature of a major cellular injury by Ni(II). In light of the activation of DNA damage responses by Ni (Fig. 1), we first examined the activation of two main genotoxic stress-sensitive kinases ATM and ATR (55, 56). We found that at doses that triggered p53 phosphorylation (Fig. 1*A*), Ni(II) also induced phosphorylation of the ATR target CHK1 kinase but produced no increases in phosphorylation of two canonical ATM targets: CHK2 and KAP1 (Fig. 2*A*). Surprisingly, inactivation of ATR or two other DNA damage-responsive kinases ATM and DNAPK by previously validated doses of their inhibitors in this cell line by us (49) did not affect the accumulation of p53 or its S15 phosphorylation by Ni(II) (Fig. 2*B*). Other stress-sensitive kinases can also activate p53 in the absence or presence of genotoxic stress. We therefore tested the involvement of three stress-activated protein kinases JNK, p38 and ERK as well as CDK9 and the energy stress-activated kinase AMPK in upregulation of p53. Inhibition of these five kinases did not produce any noticeable effects on activation of p53 by Ni(II) (Fig. S1). We then reinvestigated a potential role of ATR using a second inhibitor (VE821) which also failed to suppress p53 phosphorylation and protein accumulation by Ni(II) despite a complete loss of CHK1 phosphorylation (Fig. 2*C*). However, we noticed an unexpected effect of ATR inhibition in Ni(II)-treated cells which was activation of the DNA breaks-responsive ATM kinase as evidenced by its autophosphorylation at S1981 (Fig. 2*C*). ATR inhibition also strongly induced RPA32-S4/8 phosphorylation in Ni(II)-treated cells, indicating the accumulation of ssDNA and severe replication stress (55). A simultaneous inhibition of both ATR and ATM kinases completely eliminated p53 activation by Ni(II) (Fig. 2*D*). Thus, the initial genotoxic injury by Ni(II) activates ATR kinase that upregulates p53 and apparently prevents the formation of a more severe DNA damage sensed by ATM kinase. In agreement with its protective role, inactivation of ATR with two inhibitors strongly sensitized H460 cells to Ni(II)-induced cytotoxicity (Fig. 2*E*). Control experiments showed no significant changes in Ni(II) accumulation by ATR-inhibited cells (Fig. 2*F*). The protective role of ATR was even more dramatic for maintenance of the colony formation ability during prolonged treatments with low concentrations of Ni(II) (Fig. 2*G*). The importance of ATR in Ni(II) tolerance was further confirmed in primary human cells grown at two oxygen concentrations: 5 and 20% (Fig. 2*H*). Primary cells grew better at 5% O_2_ and showed a moderately higher sensitivity to Ni(II) cytotoxicity at these conditions. Overall, these results identified ATR as the main apical DNA damage-responsive kinase in Ni(II)-treated cells and showed that its activity was critical for the long-term cell viability and suppression of the formation of a more toxic DNA damage.

### S-phase specificity of Ni(II)-induced genotoxic stress

**Figure 3:**
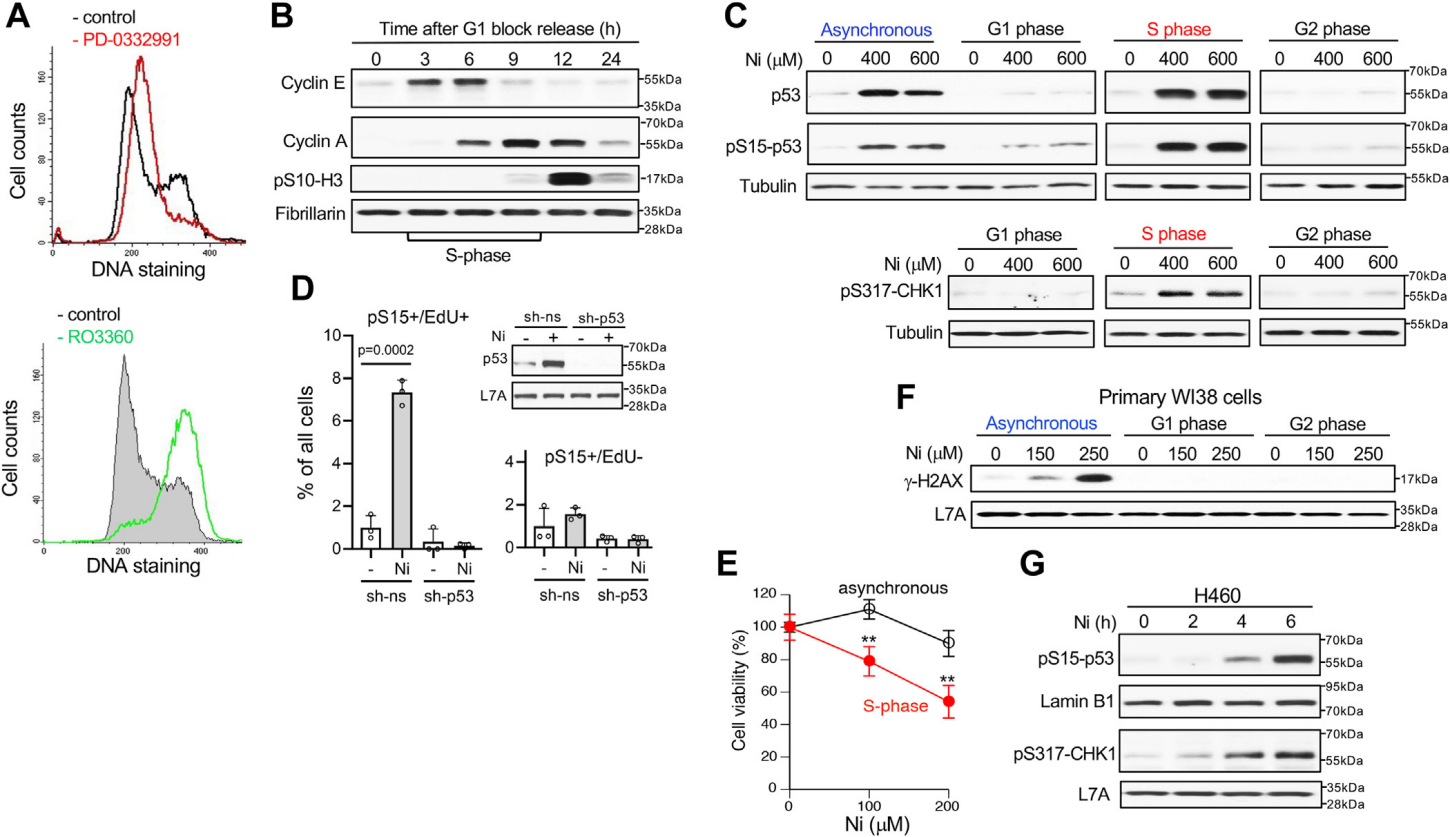
**S phase-specificity of DNA damage responses by Ni(II).** Experiments were done with H460 cells, except for panel F using primary WI38 cells. H460 cells were synchronized in G1 phase by incubations with the CDK4/6 inhibitor PD-0332991 (0.5 μM, 16 h), in G2 phase by treatments with the CDK1 inhibitor RO3360 (10 μM, 16 h) and in S phase by releasing from the G1 arrest and addition of Ni(II) at 3 h post-release. All Ni(II) treatments of H460 cells were for 6 h. *A*, FACS profiles of propidium iodide-stained control and PD0332991-treated cells (*top panel*) or control and RO3360-treated cells (*bottom panel*). *B*, progression of H460 cells through cell cycle after their release from G1 arrest. *C*, activation of p53 and CHK1-S317 phosphorylation by Ni(II) in different cell cycle phases. *D*, scoring of p53-S15 phosphorylation in EdU-positive (S-phase) and EdU-negative H460 cells treated with 0 or 400 μM Ni(II) for 6 h (sh-ns: nonspecific shRNA, sh-p53: p53-targeting shRNA). Data are means ± SD, n = 3. Insert: immunoblot demonstrating p53 knockdown by shRNA in control and Ni(II)-treated cells. *E*, hypersensitivity of S-phase cells to Ni(II) cytotoxicity. Asynchronous and S-phase-synchronized cells were treated with Ni(II) for 6 h and cell viability measurements were taken 48 h later. Data are means ± SD, n = 3, ∗∗*p* < 0.01. *F*, proliferation-dependent formation of γ-H2AX by Ni(II) in primary WI38 cells. Proliferating cells and cells arrested in G1 (24 h with 0.5 μM PD-0332991) or G2 phases (10 μM RO3360, 24 h) were treated for 24 h with Ni(II). *G*, time-dependent phosphorylation of p53 and CHK1 by 600 μM Ni(II) in H460 cells.

ATR kinase and p53 can be activated in all cell cycle phases depending on specific stressors (55, 56). To examine the phase specificity of genotoxic stress by Ni(II), we took advantage of the intact cell cycle checkpoints in H460 cells to synchronize them in different phases by targeting physiological regulators of cell cycle progression—phase-specific CDK kinases. Treatments with the CDK4/6 inhibitor PD-0332991 resulted in the accumulation of cells in the G1 phase whereas incubations with the CDK1 inhibitor RO3360 caused the arrest of cells in the G2 phase (Fig. 3*A*). G1-arrested cells readily resumed cell cycle progression after removal of the CDK4/6 inhibitor, as evident from the time-course of the buildup of the late G1/early S cyclin E and the appearance of the S/G2 cyclin A (Fig. 3*B*). The majority of cells were in S-phase at 3 to 9 h after G1 release and by 12 h they en masse entered mitosis (evidenced by abundant mitotic H3-S10 phosphorylation). Treatments with Ni(II) for 6 h in specific phases showed that activation of p53, measured by its S15 phosphorylation and protein abundance, and CHK1 phosphorylation all occurred in S phase but not in G1 or G2 phases (Fig. 3*C*). Immunostaining of asynchronous H460 populations found that p53 phosphorylation induced by Ni(II) occurred exclusively in S-phase cells (Fig. 3*D*). Specificity of phospho-p53 detection was validated by the loss of staining in Ni(II)-treated cells with p53 knockdown by shRNA. Vulnerability of replicating cells to Ni(II) injury was further evidenced by a substantially higher clonogenic lethality in H460 cells treated in S-phase *versus* asynchronous populations (Fig. 3*E*). The replication dependence of genotoxic stress by Ni(II) was further confirmed by the absence of γ-H2AX induction by Ni(II) in G1 and G2 phases of synchronized primary human cells (Fig. 3*F*). A time-course analysis of p53 and CHK1 phosphorylation showed that upregulation of these responses required at least 4-h long Ni(II) treatments and increased further after 6-h incubations (Fig. 3*G*). The timing of checkpoint signaling activation was similar to the appearance of chromatin-associated markers of genotoxic stress (Fig. 1*D*). Overall, these findings demonstrate that genotoxic stress by Ni(II) is a relatively slow developing injury that is induced primarily in S-phase of replicating cells.

### Depletion of ribonucleotide reductase subunits and dNTPs by Ni(II)

To understand a potential origin of DNA damage by Ni(II), we investigated several known replication stressors for their hyperactivation of ATM pathway in the presence of ATR inhibitors, which occurred in Ni(II)-treated cells. We initially tested formaldehyde as similar to Ni(II), this carcinogen causes chromatin remodeling and S phase-specific activation of p53 by ATR (57, 58). Formaldehyde damage to replicating chromatin was responsible for activation of ATM pathway. We found that inactivation of ATR in H460 cells with two inhibitors produced no appreciable changes in the levels of activating ATM autophosphorylation by formaldehyde (Fig. S2*A*). ATR inhibition in formaldehyde-treated primary human cells also failed to upregulate ATM as evidenced by similar phosphorylation of its targets CHK2 and KAP1 (Fig. S2*B*). Inhibition of topoisomerase I by camptothecin causes replication-dependent DNA ds-breaks and a strong activation of both ATM and ATR pathways. The addition of ATR and ATM inhibitors to H460 cells produced expected losses in phosphorylation of their targets by camptothecin (Fig. S2*C*). As observed with Ni(II), neither inhibitor eliminated p53 phosphorylation or its protein accumulation, suggesting redundant roles of these kinases in p53 activation. However, unlike Ni(II), ATR inhibition did not noticeably change CHK2 phosphorylation by ATM, which was strongly induced by camptothecin alone (also different from Ni). Treatments of ATR-inhibited cells with the DNA-crosslinking drug cisplatin led to hyperphosphorylation of CHK2 but not another ATM target—KAP1 (Fig. S2*D*). In contrast to Ni(II), p53 phosphorylation by cisplatin was severely suppressed by ATR inhibition, indicating the absence of a compensatory activation of ATM kinase toward p53. Finally, we examined responses of H460 cells to the dNTP-depleting drug hydroxyurea, which was found to be similar to Ni(II) in its ability to activate ATM (measured by KAP1 phosphorylation) only in the presence of ATR inhibitors (Fig. S2*E*). Also as found for Ni(II), p53 phosphorylation by hydroxyurea was not suppressed by ATR inactivation.

**Figure 4:**
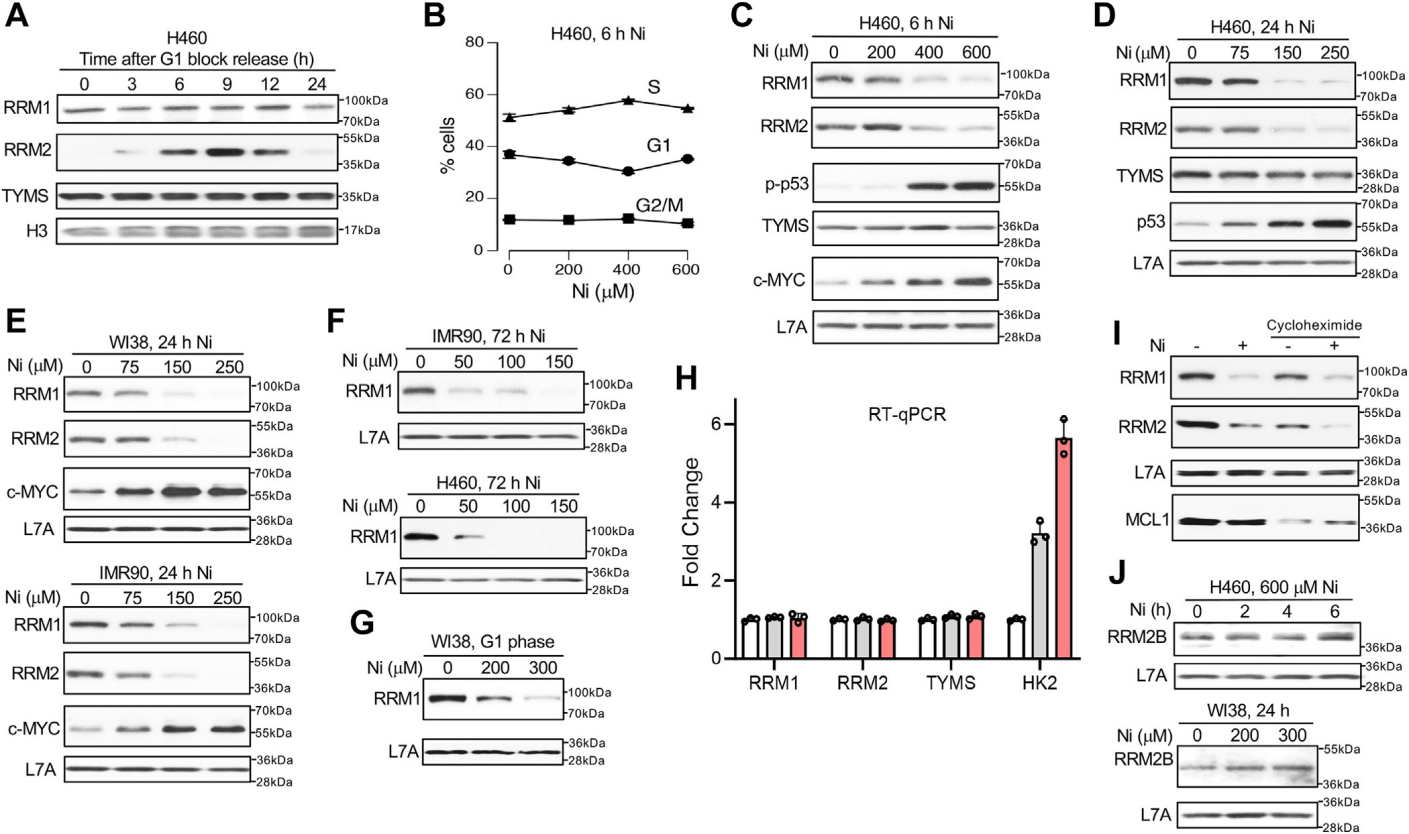
**Loss of ribonucleotide reductase subunits in Ni(II)-treated cells.** Whole-cell lysates were used for all Westerns. *A*, abundance of dNTP-producing proteins during cell cycle progression. H460 cells were arrested in the G1 phase by incubation with 0.5 μM PD-0332991 for 16 h and then collected for westerns at different times after release from the G1 block. *B*, distribution of H460 cells among different cell cycle phases following 6 h treatments with Ni(II). Data are means ± SD, n = 2. *C*, immunoblots of H460 cells treated with Ni(II) for 6 h or (*D*) 24 h. *E*, RRM1, RRM2 and c-MYC abundance in primary human cells treated with Ni(II) for 24 h. *F*, RRM1 levels in H460 and primary IMR90 cells after 72 h treatments with Ni(II). *G*, RRM1 abundance in G1 phase-arrested primary WI38 cells treated with Ni(II) for 24 h. Cells were incubated with 0.5 μM PD-0332991 for 24 h followed by the incubation with Ni(II) in the presence of the G1 phase blocker. *H*, mRNA levels measured by RT-qPCR in H460 cells treated with Ni(II) for 6 h. Means ± SD, n = 3. *I*, RRM1 and RRM2 depletion in H460 cells by 600 μM N(II) treatments for 6 h in the absence and presence of cycloheximide (100 μg/ml). A loss of a short-lived protein MCL1 serves as a biomarker of protein synthesis inhibition. *J*, immunoblots for RRM2B in Ni(II)-treated H460 and WI38 cells.

The observed close similarity in DNA damage responses of hydroxyurea- and Ni(II)-treated cells under ATR-inhibited conditions prompted us to investigate Ni(II) effects on dNTP-producing enzymes. The entire *de novo* biosynthesis of dNTPs from ribonucleotides is controlled by the sole cellular enzyme ribonucleotide reductase (RNR) which is a target of hydroxyurea (59, 60). Thymidylate synthase (TYMS) is necessary for methylation of dUMP to produce dTMP. The proliferation-promoting transcription factor c-MYC is known to stimulate expression of nucleotide biosynthesis genes but not RNR subunits RRM1 and RRM2 (61). Similar to other cells (59), RRM2 expression in H460 cells was practically undetectable in G1 phase, dramatically upregulated in the S phase, and then decreased in G2 phase (Fig. 4*A*), providing a physiological validation of RRM2 specificity of the employed antibody. Also as observed in other cells, RRM1 and TYMS were expressed constantly throughout the cell cycle in H460 cells. Treatments with Ni(II) for 6 h did not cause major changes in cell cycle distribution (Fig. 4*B*), allowing us to examine the protein abundance of RRM2 without significant phase-dependent effects. The absence of marked cell cycle changes at this time is consistent with the initial activation of checkpoint signaling between 4 to 6 h Ni(II) (Fig. 3*G*) and its insufficient duration to cause a major redistribution of cells among different phases. A strong upregulation of HIF1α by Ni(II) (shown in Fig. 1*A*) is known to cause a global slowdown of cell cycle progression (62, 63), making Ni(II)-treated populations slowly responsive to cell cycle changes after activation of checkpoint signaling. We found that 6 h Ni(II) treatments induced large losses of both RRM1 and RRM2 subunits of RNR, which closely paralleled the dose dependence in p53 phosphorylation (Fig. 4*C*). There were no apparent changes in the protein abundance of TYMS in Ni(II)-treated cells whereas c-MYC levels were strongly elevated in a dose-dependent manner. Longer treatments with Ni(II) for 24 h also led to the loss of both RNR subunits, which inversely followed the dose dependence of p53 accumulation (Fig. 4*D*). The levels of TYMS remained unchanged as seen in shorter treatments.

Next, we examined levels of dNTP-producing enzymes in two primary human cell lines. Similar to H460 cells, both primary lines showed steep decreases in abundance of RRM1 and RRM2 after 24 h Ni(II) treatments whereas c-MYC levels were clearly elevated (Fig. 4*E*). The depletion of RRM2 was not caused by the loss of S-phase cells in Ni(II)-treated primary IMR90 cells as measured by a two-parameter FACS assay (EdU incorporation and DNA staining): 26.2 ± 0.6% control cells in S-phase *versus* 27.2 ± 1.2% Ni-treated cells in S phase (not significant differences, n = 3). However, Ni(II) caused significant changes in the size of G1 and G2 populations: controls had 56.8 ± 1.7% *versus* 50.9 ± 0.9% Ni-treated cells in G1 phase (*p* < 0.01, n = 3) and 14.9 ± 0.8% *versus* 19.8 ± 1.6% in G2 phase (*p* < 0.01, n = 3). A larger size of the G2 population in Ni(II)-treated samples probably reflects a continuing activity of the replication stress-initiated checkpoint signaling (inhibiting G2-M progression) and some remaining DNA damage even after the progression of cells from S into G2. Prolonged 72 h Ni treatments were even more effective in depleting normally stable RRM1 in H460 and primary cells (Fig. 4*F*). In comparison to 24 h, 72 h incubations required ∼3 times lower concentrations of Ni(II) to cause comparable losses of RRM1. After 50 μM treatments of H460 cells, cellular concentrations of Ni were only 4 ± 0.5 μM (n = 3), indicating a high vulnerability of RRM1 at low internal doses of Ni in chronic exposures. The depletion of RRM1 by Ni(II) was independent of its dimerization partner RRM2, as evidenced by the loss of RRM1 in G1-arrested cells (Fig. 4*G*) which do not express RRM2 as shown in Figure 4*A*.

To explore potential mechanisms for RRM1 and RRM2 losses in Ni(II)-treated cells, we measured their mRNA levels by RT-qPCR. We found no significant changes in the expression of both RNR genes or thymidine-producing TYMS (Fig. 4*H*). As expected, Ni(II) caused a dose-dependent increase in transcription of the HIF1-inducible HK2, demonstrating the technical proficiency of our assay. Ni(II) retained its ability to deplete RRM1 and RRM2 in the presence of the protein synthesis inhibitor cycloheximide (Fig. 4*I*), indicating that the loss of both proteins was caused by their faster turnover/destabilization. Parallel immunoblots with our main set and a second pair of antibodies against RRM1 and RRM2 showed practically identical losses of both proteins in Ni(II)-treated cells (Fig. S3*A*), arguing against antibody-specific effects in our findings. The specificity of both sets of antibodies against RRM1 and RRM2 was confirmed using spiked samples, siRNA knockdown, and physiological validation through analysis of cell cycle-dependent expression of RRM2 (Fig. S3, *B*–*D*). RRM2-RRM1 heterodimer is a main form of RNR generating dNTPs in replicating cells. In G1 phase where RRM2 is not expressed, a complex of RRM1 with a less abundant isoform RRM2B serves as the source of dNTPs for genome maintenance (59, 60). RRM2B is a p53-inducible protein and its expression is frequently increased under stress. We found that in contrast to RRM2 depletion but in agreement with p53 activation, RRM2B protein abundance was increased in Ni-treated cells, which was more noticeable for 24 h treatments in primary WI38 cells (Fig. 4*J*). A relatively moderate increase in RRM2B protein levels in H460 cells treated for 6 h reflects slow kinetics of p53 activation by Ni(II) (Fig. 3*G*). It is possible that upregulated RRM2B partially compensates for the loss of RRM2.

**Figure 5:**
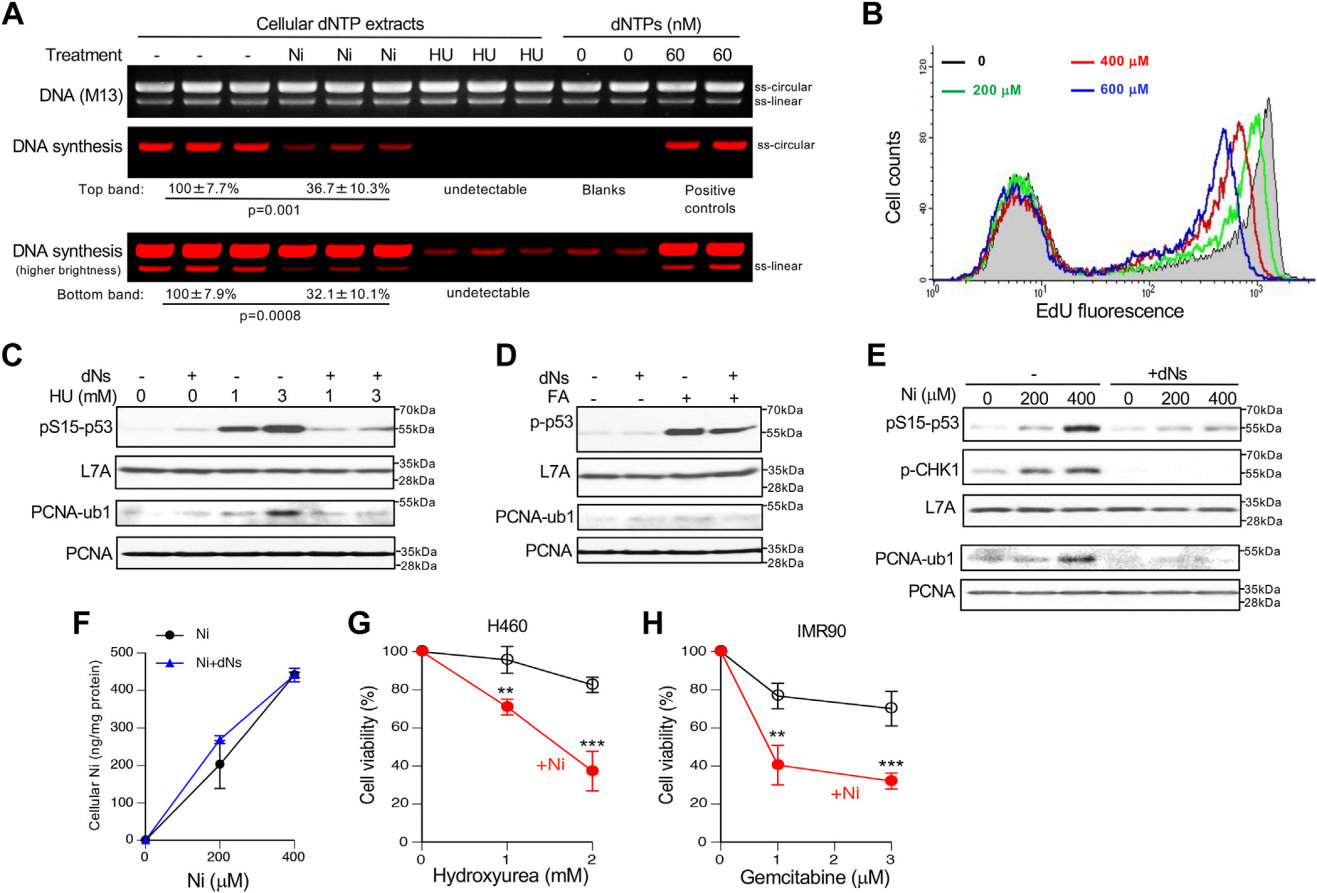
**Role of impaired ribonucleotide reductase activity in DNA damage responses to Ni(II).** H460 cells were treated for 3 h with hydroxyurea (HU) or formaldehyde (FA) and for 6 h with Ni(II). *A*, measurements of cellular levels dNTPs using primer extension reactions on M13 ss-DNA. DNA synthesis was detected by the incorporation of fluorescent CF680R-dUMP. Cells were treated with 5 mM hydroxyurea for 3 h or 600 μM Ni(II) for 6 h and three independent samples of dNTP extracts were prepared for each condition. *B*, representative FACS profiles of EdU-labeled H460 cells treated with Ni(II) for 6 h. EdU was added during the last hour of Ni(II) treatments. *C*, elimination of p53 phosphorylation and PCNA monoubiquitination (PCNA-ub1) by hydroxyurea in the presence of exogenous deoxyribonucleosides (dNs). *D*, no effects of exogenous dNs on p53 phosphorylation and PCNA monoubiquitination by formaldehyde (FA, 200 μM). *E*, loss of DNA damage responses to Ni(II) in the presence of exogenous dNs. *F*, cellular accumulation of Ni(II) in the absence or presence of exogenous dNs. Means ± SD, n = 3. *G*, viability of H460 cells cotreated with Ni (0 or 400 μM) and hydroxyurea for 6 h. Cell viability measurements were taken after 48 h recovery. Means ± SD, n = 4, ∗∗*p* < 0.01, ∗∗∗*p* < 0.001. *H*, viability of primary IMR90 cells cotreated with gemcitabine and Ni (0 or 150 μM) for 48 h. Cell viability measurements were taken after 24 h recovery. Means ± SD, n = 4, ∗∗*p* < 0.01, ∗∗∗*p* < 0.001.

Downregulation of RNR subunits, especially a rate-limiting inducible RRM2, indicates that Ni(II)-treated cells could experience a deficient production of dNTPs. We found approximately a 3-fold reduction in the pool of cellular dNTPs after 6 h treatments with Ni(II) (Fig. 5*A*). The extent of the dNTP depletion was moderately weaker than the loss of RRM2 for the same Ni(II) dose (remaining RRM2: 17 ± 7.3%, n = 4), which likely reflects some compensatory effects of elevated RRM2B and activation of S-phase checkpoint slowing down replication and consequently, diminishing utilization of dNTPs. Insensitivity of our measurements of dNTPs to the presence of more abundant rNTPs in cellular extracts was evident from the absence of detectable increases in DNA synthesis in samples from RNR-inhibited cells (hydroxyurea lanes) relative to blanks. We calculated that the average concentration of measured three dNTPs (dATP, dCTP, dGTP) was 9.8 ± 0.8 pmol/10^6^ H460 cells, which is close to the average values found for the same three dNTPs in other transformed cells (7–8 pmol/10^6^ K562 and 293T cells) (64). Measurements of DNA synthesis by FACS using EdU incorporation showed a dose-dependent inhibition of DNA replication (Fig. 5*B*) with the extent of inhibition by 600 μM Ni(II) being comparable to the measured reduction in the cellular pool of dNTPs. It is possible that inhibitory effects of Ni(II) on DNA synthesis were somewhat underestimated as the decrease in the pool of cellular dTTP due to a diminished activity of RNR should increase DNA incorporation of EdU due to a lower competition from a normal nucleotide. To test the role of impaired dNTP production in Ni(II)-induced DNA damage responses more directly, we conducted rescue experiments by providing cells with exogenous deoxyribonucleosides (dNs), which bypasses the requirement for RNR activity to produce dNTPs in cells. As expected, supplementation of cells with exogenous dNs abolished p53 phosphorylation and the formation of monoubiquitinated PCNA by the RNR inhibitor hydroxyurea (Fig. 5*C*). Induction of ATR-mediated p53 phosphorylation by formaldehyde, which occurs as a result of the stalling of replicative helicases by DNA-protein crosslinks (57), was not affected by the addition of dNs (Fig. 5*D*). Consistent with the lack of a direct blockage of elongating DNA polymerases, formaldehyde did not induce monoubiquitination of PCNA in the absence or presence of exogenous dNs. Having validated the specificity of the rescue effects by exogenous dNs, we next examined the impact of their addition on Ni(II)-induced DNA damage. We found that media supplementation with dNs eliminated activation of ATR kinase as evidenced by the loss of phosphorylation of its two targets: p53 and CHK1 (Fig. 5*E*). Exogenous dNs also abolished PCNA monoubiquitination by Ni(II), indicating the loss of DNA polymerase-blocking effects. Importantly, dNs did not cause any significant changes in the cellular accumulation of Ni(II) (Fig. 5*F*). The biological significance of RRM1/RRM2 losses by Ni(II) was further demonstrated by a strong sensitization of Ni(II)-treated H460 and primary human cells to cytotoxicity of two clinically used RNR inhibitors - hydroxyurea and gemcitabine (Fig. 5, *G* and *H*).

### FL-RIB assay for detection of DNA-incorporated rNMPs

**Figure 6:**
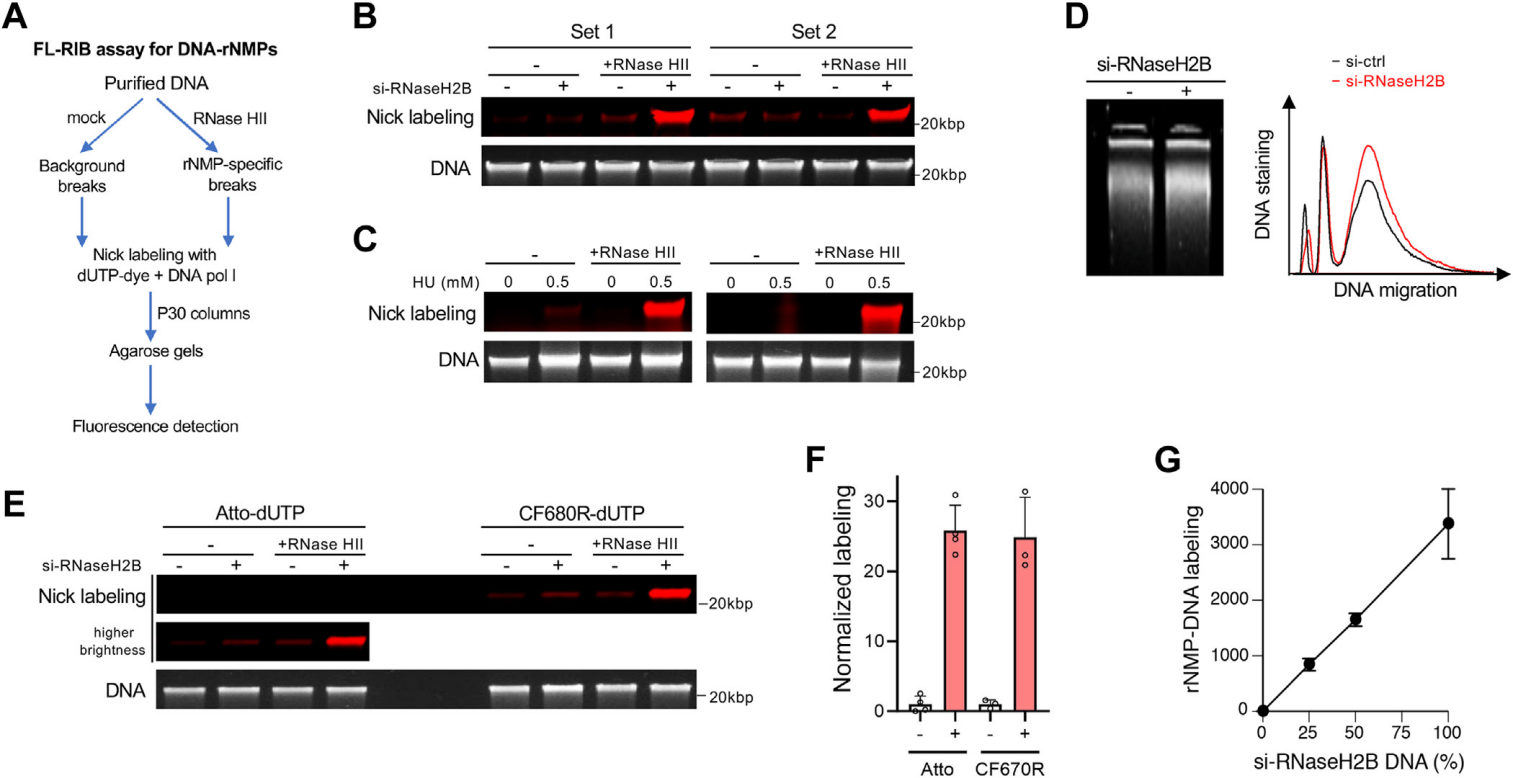
**FL-RIB assay for detection of DNA-embedded rNMPs.***A*, flow chart of the FL-RIB assay. *B*, detection of DNA-incorporated ribonucleotides in control and RNaseH2B-depleted H460 cells using Atto-dUTP. Results of labeling reactions for two sets of independent DNA samples (320 ng each reaction) are shown. High purity of Atto-dUTP in this experiment allowed skipping a pre-electrophoresis purification of DNA by P30 columns. *C*, detection of DNA-incorporated dNMPs in hydroxyurea-treated H460 cells (0.5 mM HU, 24 h) with Atto-dUTP labeling of RNaseHII-induced breaks. A complete FL-RIB protocol with DNA purification by P30 columns was followed. Agarose gels were run with 300 ng DNA. *D*, representative agarose gel of alkali-treated DNA and a scan of gel fluorescence after SYBR Gold staining. DNA samples from control and RNaseH2B-depleted H460 cells were treated with alkali (0.3 mM NaOH for 2 h, 55 °C) and ran on 1% agarose alkaline gel (50 mM NaOH, 1% EDTA). *E*, comparison of the FL-RIB assay using Atto-dUTP *versus* CF680R-dUTP. A standard protocol with P30 spin column purification for all reactions was followed. DNA samples isolated from control and RNaseH2B knockdown cells were tested. *F*, fold increase in DNA labeling by FL-RIB using Atto-dUTP (means ± SD, n = 4) or CF680R-dUTP (means ± SD, n = 3). *G*, dose-dependence of DNA-rNMP detection by the FL-RIB assay using CF680R-dUTP (means ± SD, n = 2). Samples with a different mix of DNA from control and RNaseH2B knockdown cells were tested for DNA labeling by the FL-RIB assay. Y-axis shows the fluorescence of DNA-incorporated dUTP after background subtraction (control DNA only).

A shortage of dNTPs leads to higher incorporation of rNMPs into DNA due to imperfect discrimination between dNTPs and rNTPs by all major DNA polymerases (6, 7). Removal of DNA-incorporated rNMPs is primarily performed by ribonucleotide excision repair (RER) that is initiated by RNase H2 through the incision at the DNA-RNA junction generating a single-stranded DNA break containing 3′-OH and 5′-phosphate ends (65). DNA polymerase δ or ε then adds nucleotides to the 3′-OH side, creating a flap containing the rNMP. This excessive DNA is removed by the flap endonuclease FEN1 or by the exonuclease EXO1. Sealing of the remaining DNA nick by a DNA ligase completes the repair process (65). ^32^P-dNTP-based labeling of RNaseHII-generated 3′-OH ends in purified DNA was used for the determination of DNA-incorporated rNMPs (10). Based on the same principle, we developed a sensitive fluorescent method for measurements of ribonucleotides in DNA – FL-RIB (Fluorescent Labeling of RNaseHII-Induced Breaks) assay (Fig. 6*A*). Notable features of this assay are (1) the use of fluorescent dUTP derivatives for detection in the low-background near-infrared region, (2) a single-tube digestion/labeling procedure (which eliminates risks of DNA losses and inhibitory effects of residual ethanol in the original radioactive procedure) and (3) small amounts of DNA needed for analysis (0.1–0.5 μg for Atto-dUTP and even less for CF680R-dUTP reactions). We first tested the ability of this assay to detect rNMPs in cellular DNA by comparing samples from control and RNaseH2-depleted cells (Fig. 6*B*). Two independent sets of samples showed a highly increased Atto680-dUTP labeling of DNA from cells with inactivated RER (RNaseH2B knockdown) when these DNA samples were treated with recombinant RNase HII. As expected for dNTP-deficient conditions, highly elevated levels of rNMPs were also detected in DNA from cells treated with the RNR inhibitor hydroxyurea (Fig. 6*C*). DNA size profiling after treatment with alkali is currently the most frequently used methodology for the detection of excessive presence of rNMPs (66, 67, 68). This approach is suitable for the detection of large amounts of DNA-incorporated rNMPs but it is not very sensitive. Agarose electrophoresis of alkali-treated DNA showed increased amounts of smaller DNA fragments in samples from RNaseH2B knockdown cells relative to control cells (Fig. 6*D*). The magnitude of the alkali-induced increase in DNA fragmentation was modest in comparison to dramatic differences detected for the same pair of cells by the FL-RIB assay (Fig. 6, *B*, *E* and *F*). Testing of the FL-RIB assay with another dUTP derivative (CF680R-dUTP) showed that it produced a much greater fluorescence (14.1-fold) in DNA labeling reactions in comparison to Atto-dUTP (Fig. 6*E*). However, when the results of DNA-rNMP measurements in RNaseH2B knockdown cells were normalized to control cells, both dUTP conjugates yielded similar (∼25-fold) increases (Fig. 6*F*). Thus, these two dUTP derivatives offer a similar relative sensitivity but CF680R-dUTP is better suited for samples with smaller amounts of DNA (<100 ng) and other conditions with weaker labeling or less sensitive detectors. Samples of CF680R-dUTP consistently contained high levels of fluorescent impurities some of which comigrated with DNA on agarose gels which made it necessary to pass labeled DNA through P30 columns prior to loading on gels. For Atto-dUTP, P30 column purification was required for some but not all batches. Finally, we tested the FL-RIB assay for the dose dependence and found linear responses in DNA labeling *versus* amounts of DNA-incorporated rNMPs (Fig. 6*G*).

### DNA-incorporated rNMPs in Ni(II) genotoxicity

**Figure 7:**
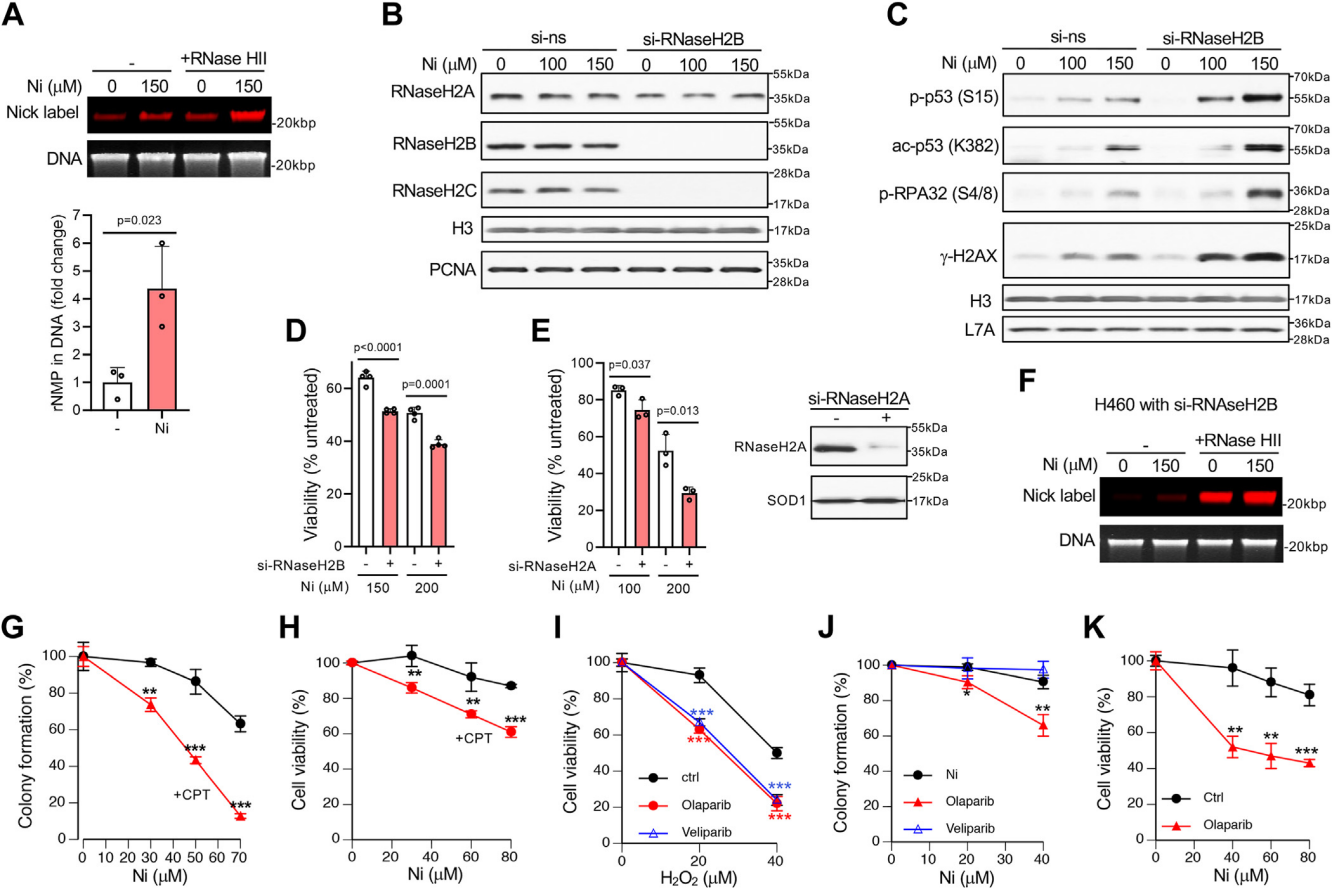
**Enhanced genotoxicity of DNA-embedded rNMPs by Ni(II).***A*, rNMP detection in DNA from control and Ni(II)-treated H460 cells (24 h Ni) and data quantitation for three independent DNA samples in each group (means ± SD). FL-RIB assay for DNA-rNMPs was done with Atto-dUTP. *B*, protein levels of RNase H2 subunits in H460 cells transfected with control or RNaseH2B-targeting siRNA and then treated with Ni(II) for 24 h. *C*, DNA damage responses in cells treated as in *panel B*. *D*, viability of control and RNaseH2B-depleted H460 cells treated with Ni(II) for 18 h and assayed after 24 h recovery (means ± SD, n = 4). *E*, viability of control and RNaseH2A-depleted WI38 primary cells treated with Ni(II) for 48 h and assayed after 24 h recovery (means ± SD, n = 3). *Right panel*: immunoblot demonstrating RNaseH2A knockdown in WI38 cells. *F*, FL-RIB assay for DNA-embedded rNMPs in RNaseH2B-depleted H460 cells treated with Ni(II) for 24 h. *G*, colony formation by H460 cells treated with Ni(II) in the presence of 3 nM camptothecin (CPT) for 8 days. Means ± SD, n = 3, ∗∗*p* < 0.01, ∗∗∗*p* < 0.01. *H*, viability of primary WI38 cells treated with Ni(II) in the presence of 5 nM camptothecin (CPT) for 5 days. Means ± SD, n = 4, ∗∗*p* < 0.01, ∗∗∗*p* < 0.01. *I*, viability of H460 cells treated with H_2_O_2_ for 48 h in the presence of 3 μM PARP inhibitors olaparib and veliparib. Means ± SD, n = 4, ∗∗∗*p* < 0.001. *J*, colony formation by H460 cells treated with Ni(II) in the presence of 2 μM olaparib or veliparib. Means ± SD, n = 3, ∗*p* < 0.05, ∗∗*p* < 0.01. *K*, viability of primary IMR90 cells treated with Ni(II) for 120 h in the presence of 3 μM olaparib. Means ± SD, n = 3, ∗∗*p* < 0.01, ∗∗∗*p* < 0.001.

Stimulation of rNMP incorporation into DNA by the RNR inhibitor hydroxyurea prompted us to investigate whether Ni(II) also increases the DNA burden of genotoxic ribonucleotides. Analysis of DNA preparations from Ni(II)-treated cells by the FL-RIB assay found a clearly elevated presence of DNA-rNMPs, which was statistically significant (Fig. 7*A*). The signal from background DNA breaks with 3′-OH ends, which was subtracted in the calculations of RNase HII-induced specific labeling (rNMPs), did not differ between control and Ni(II) treated samples (100 ± 29.8% in controls *versus* 113 ± 19.5% for Ni, n = 3). RNase H2 is responsible for the initiation of the RER-mediated removal of DNA-embedded rNMPs through the incision at the DNA-RNA junction (65). Next, we examined DNA damage responses to Ni(II) in cells with compromised ribonucleotide excision repair *via* RNase H2 knockdown. RNase H2 is a heterotrimeric nuclease composed of three subunits: catalytic RNaseH2A and regulatory RNaseH2B and RNaseH2C. We inactivated RNase H2 by targeting its RNaseH2B subunit with siRNA and evaluated DNA damage responses after 24-h long treatments of H460 cells with Ni(II) doses that gradually deplete RNR subunits (Fig. 4, *D* and *E*). This treatment duration encompasses at least two S-phase passages for H460 cells. Knockdown of RNaseH2B also caused a co-depletion of its binding partner RNaseH2C, indicating its instability when not incorporated into the trimeric RNaseH2 complex (Fig. 7*B*). Ni(II) alone did not cause marked changes in the abundance of any of the three RNase H2 subunits. A loss of RNase H2 strongly increased DNA damage by Ni(II), as evidenced by markedly elevated levels of several markers of genotoxic stress such as S15-phosphorylated p53, phosphorylated RPA32 (S4/S8 sites) and γ-H2AX (Fig. 7*C*). RNase H2 knockdown also enhanced the acetylation of p53 at its C-terminal domain (measured at K382 site), which is important for the transactivation of a majority of p53-regulated genes (69). Consistent with the observed enhancement of DNA damage responses, Ni(II) was also more cytotoxic in H460 cells with RNaseH2B knockdown (Fig. 7*D*) and in primary WI38 cells with knockdown of a different subunit of RNase H2 – RNaseH2A (Fig. 7*E*). However, Ni(II) treatments of RNaseH2B-depleted H460 cells did not produce marked changes in the already very high levels of DNA-incorporated rNMPs (Fig. 7*F*). The lack of apparent differences between control and Ni-treated RER-deficient cells probably reflects lower spontaneous incorporation of rNMPs into DNA due to replication-inhibitory effects of Ni(II) treatments, which would neutralize a heightened contribution of Ni-induced rNMPs to their overall burden in DNA. We also cannot exclude the possibility that our assay is less responsive at >25× normal levels of DNA-rNMPs detected in RNaseH2B-depleted cells without Ni treatments (Fig. 6*F*).

Treatments of H460 cells with 150 μM Ni(II) caused an approximately 4-fold increase in DNA-rNMPs (Fig. 7*A*) and clearly detectable DNA damage responses in cells with a nonspecific siRNA (Fig. 7*C*). However, a much larger burden of rNMPs in DNA of RNase H2B-depleted cells without Ni(II) treatments produced practically no detectable increases in markers of genotoxic stress in parallel analyses, indicating that although DNA-embedded rNMPs are genotoxic in chronic models of RER deficiency (11, 18), they are not acutely genotoxic and their modest formation by Ni(II) cannot be directly responsible for its genotoxic effects. Elevated genotoxicity and cytotoxicity of Ni(II) in RNaseH2 knockdowns suggest that Ni(II) could enhance the toxicity of DNA-rNMPs during their processing by an alternative removal process that involves topoisomerase I (Top1). DNA-embedded rNMPs are known as a major source of covalently DNA-trapped TOP1 at 3′-phosphate of DNA ss-break (70). Unrepaired TOP1-DNA crosslinks (DNA-protein crosslinks, DPC) are highly toxic due to their interference with normal DNA functions and conversion into DNA ds-breaks (71). To test whether Ni(II) enhances the cytotoxicity of Top1-DPC, we examined the viability of human cells chronically treated with low doses of Ni(II) and the Top1-DPC-forming drug camptothecin (CPT). We found that treatments of H460 and primary WI38 cells with Ni(II) made them hypersensitive to CPT (Fig. 7, *G* and *H*). DNA cleavage by Top1 in the vicinity of embedded rNMPs generates DNA ss-breaks that are bound by PARP to initiate their repair. Trapping of PARP in the DNA break-bound but inactive state by structural PARP inhibitors such as olaparib, but not inhibition of PARP activity by the catalytic inhibitor veliparib, results in more toxic DNA lesions (72). Consistent with the canonical role of PARP in base excision repair of oxidative damage, we found that PARP inhibitors olaparib and veliparib produced similar decreases in viability of H_2_O_2_-treated H460 cells (Fig. 7*I*). However, only the DNA-trapping drug olaparib but not veliparib decreased long-term survival after low-dose Ni(II) exposures in H460 cells (Fig. 7*J*). Olaparib also significantly enhanced the toxicity of Ni(II) in primary human cells (Fig. 7*K*). Overall, our findings showed Ni(II) caused genotoxic effects associated with the presence of DNA-embedded rNMPs primarily *via* their more toxic processing and additionally, by increasing their abundance.

### Impairment of SUMO-dependent repair of TOP1-DPC by Ni(II)

**Figure 8:**
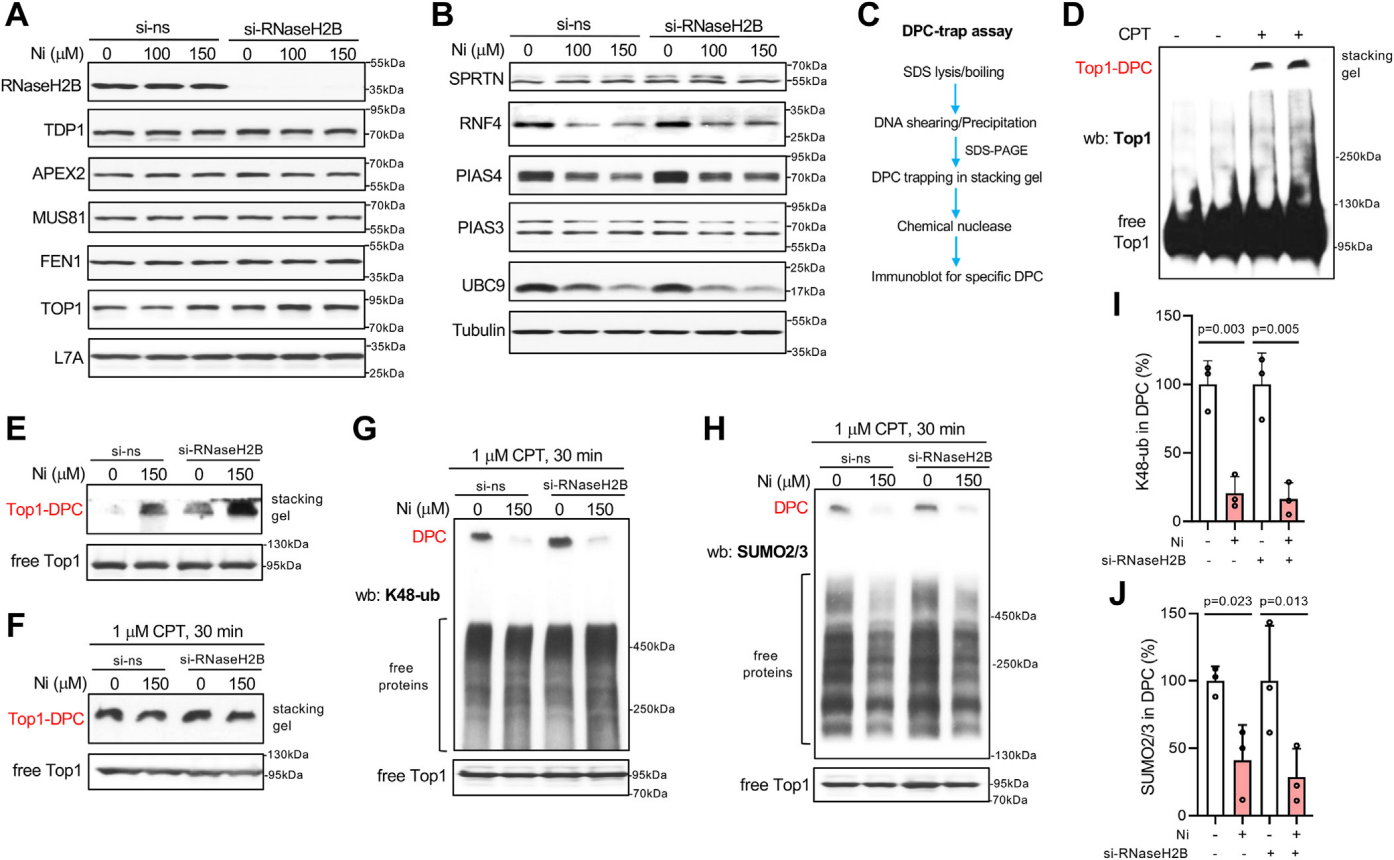
**Impaired processing of Top1-DPC in Ni(II)-treated H460 cells.***A* and *B*, immunoblots for proteins involved in the processing of Top1-DPC. *C*, flow chart depicting major steps in the DPC-trap assay. *D*, Top1 immunoblot demonstrating detection of both DPC and free Top1 in H460 cells treated with 1 μM CPT for 1 h. *E*, Top1-DPC in H460 cells treated with Ni(II) for 18 h. Free Top1 band is taken from the same blot but after a shorter exposure. *F*, Top1 immunoblot for Top1-DPC in H460 cells treated with Ni(II) for 18 h and then for 30 min with 1 μM CPT. Free Top1 band is taken from the same blot but after a shorter exposure. *G*, detection of K48-polyubiquitination in Top1-DPC and free cellular proteins. The same samples were used as in *panel F*. *H*, detection of polySUMOylation (SUMO2/3) in Top1-DPC and free cellular proteins. The same samples were used as in *panels F* and *G*. *I*, quantitation of K48-polyubiquitination in Top1-DPC shown in *panel G* (means ± SD, n = 3). *J*, quantitation of polySUMOylation in Top1-DPC shown in *panel H* (means ± SD, n = 3).

Increased geno- and cytotoxicity of TOP1-DPC in Ni(II)-treated cells resembled the phenotype of cells with defects in the normal repair of these lesions, leading to a switch to their cleavage by flap endonucleases, especially by MUS81 (73). The observed above depletion of RRM subunits led us to consider the possibility that some other nuclear proteins such as those involved in TOP1-DPC removal could be also downregulated by Ni(II). Immunoblots for enzymes involved in the removal of residual TOP1-peptide DPC [TDP1, APEX2(APE)] and nucleases implicated in a more genotoxic pathway for TOP1-DPC removal (MUS81, FEN1) showed no marked changes in their protein expression (Fig. 8*A*). The initial step in the repair of TOP1-DPC prior to the action of TDP1 and APEX2 involves their debulking *via* direct proteolysis by SPRTN and/or by a polySUMO-dependent proteasomal degradation (71). We found no apparent changes in the expression of SPRTN protease whereas protein levels of three enzymes (RNF4, PIAS4, and UBC9) involved in the SUMO-dependent proteolysis of TOP1-DPC were clearly decreased by Ni(II) (Fig. 8*B*). PIAS4 is the E3 SUMO ligase acting on TOP1-DPC (74), UBC9 is the sole E2 SUMO ligase and RNF4 is the polySUMO-dependent E3 ubiquitin ligase for TOP1-DPC (74). The abundance of PIAS3, another E3 SUMO-conjugating enzyme that is not involved in DPC repair, was not changed in response to Ni(II).

Downregulation of RNF4, PIAS4, and UBC9 can be expected to lead to impaired polySUMOylation and removal of TOP1-DPC in Ni(II)-treated cells. To investigate these possibilities, we developed a new assay (DPC-trap assay) for specific and sensitive detection of TOP1-DPC and their modifications by SUMO or ubiquitin (Fig. 8*C*). The specificity of the assay relies on the use of immunodetection and a very clean separation of very bulky DPC (>10 MDa for typical >20 kbp DNA fragments) and free TOP1 (91 kDa) by SDS-PAGE. The whole cell lysates are run on the gels permitting easy testing for equal sample loading (free TOP1) and assessment of modifications in free proteins *versus* those in DPC. The very large size of DPC severely inhibits their transfer to membranes for immunoblotting. We solved this problem by cleavage of DNA in the gel-trapped DPC by a small, gel-penetrating chemical nuclease composed of the DNA-binding complex of bleomycin-Fe(III) activated by ascorbate (75), which then allows a standard transfer of DNA-released TOP1 for immunoblotting. A test experiment with TOP1-DPC induced by CPT showed a strong signal for DPC and their clear separation from free TOP1 (Fig. 8*D*). The separation between DPC and TOP1 or other free proteins can be easily increased further (as shown in Fig. 8, *G* and *H*) by running gels longer. Using the DPC-trap assay, we found that control cells treated with Ni(II) for 18 h had elevated levels of TOP1-DPC, which were increased even more by RNaseH2B knockdown (Fig. 8*E*), correlating well with the observed above genotoxic responses (Fig. 7*C*). To investigate the initial steps in the proteolytic repair of TOP1-DPC, we formed a large number of TOP1-DPC by a short treatment with CPT of control and Ni(II)-treated cells with and without RNaseH2B depletion. As expected, under these conditions the levels of TOP1-DPC induced by CPT were similar among all groups (Fig. 8*F*). Analysis of the same CPT-treated samples by the DPC-trap assay using immunoblotting for K48-linked polyubiquitin found severely depressed levels of this proteolytic modification in TOP1-DPC from Ni(II)-treated cells whereas polyubiquitination of free proteins was largely unchanged or even slightly higher (Fig. 8*G*). Quantitation of data from three biological replicates confirmed a highly significant, several-fold decrease in TOP1-DPC ubiquitination by Ni(II) in both control and siRNaseH2B-depleted cells (Fig. 8*I*). Because of the downregulation of proteins involved in the ubiquitination-promoting DPC SUMOylation (RNF4, UBC9, and PIAS4), we next examined the extent of SUMOylation of the same CPT-induced TOP1-DPC that showed severely depressed K48 polyubiquitination by Ni(II). Consistent with the depletion of SUMOylation enzymes, we found that Ni(II) strongly decreased SUMOylation of TOP1-DPC (Fig. 8, *H* and *J*). SUMOylation of free cellular proteins was also noticeably decreased by Ni(II), most likely reflecting lower levels of UBC9 which is the sole SUMO E2 ligase in human cells. Overall, these findings showed that Ni(II) impairs proteolytic repair of TOP1-DPC by inhibiting their SUMOylation and the subsequent formation of proteasome-recruiting K48-linked polyubiquitin by RNF4. In addition to low SUMOylation, the downregulation of RNF4 by Ni(II) further exacerbates K48 polyubiquitination defects of TOP1-DPC.

## Discussion

A widespread environmental pollutant Ni(II) has long been described as a nongenotoxic carcinogen due to its inability to react with DNA and induce significant mutagenic responses in the standard bacterial and mammalian test systems (38, 39, 40). The results of our work here provide strong evidence for indirect Ni(II) genotoxicity resulting from a previously unsuspected mechanism. Our investigation of upstream stress signaling mechanisms leading to the upregulation of the transcription factor p53 by Ni(II) uncovered the ability of this carcinogenic metal to cause replication stress and trigger S phase-specific activation of the apical DNA damage-induced kinase ATR. Ni(II)-treated cells showed a strong dependence on ATR activity for suppression of severe replication stress and their long-term survival. Destabilization of both subunits of the dNTP-producing ribonucleotide reductase and the resulting dNTP deficiency were the primary causes of replication stress and replication-associated DNA damage by Ni(II) in short-term exposures. In prolonged treatments, impairment of dNTP biosynthesis by Ni(II) also led to a significantly increased misincorporation of rNMPs into DNA. Overall genotoxic effects detected in Ni(II)-treated cells were similar to those found in mammalian genetic models with chronically increased amounts of DNA-embedded rNMPs (9, 10, 11). However, the observed increases in DNA-rNMPs of Ni-treated cells were insufficient to directly cause noticeable DNA damage responses in subchronic exposures but their genotoxicity was strongly increased by a severely impaired SUMO-dependent proteolytic repair of TOP1-DPC that is primarily caused by unrepaired DNA ribonucleotides (70). This repair defect resulted from the Ni-induced downregulation of DPC-SUMOylating E2 (UBC9) and E3 (PIAS4) enzymes as well as the SUMO-dependent E3 ubiquitin ligase RNF4. Thus, selective proteotoxicity by Ni(II) appears to be primarily responsible for the main causes of its genotoxicity: dNTP deficiency, elevated DNA-rNMPs and impaired repair of TOP1-DPC frequently formed at the sites of DNA-embedded ribonucleotides. Interference with the functions of metalloproteins through competitive replacement of Fe^2+^, Zn^2+^, or Mg^2+^ represents another potential route for DNA repair inhibition by Ni^2+^ ions (35, 36, 37).

Cytogenetic studies with Ni(II) in cultured cells (41, 42, 43) and among Ni(II)-exposed workers (76, 77) all found chromosomal aberrations. Based on its nonmutagenicity and DNA unreactivity, chromosomal abnormalities by Ni(II) have been historically attributed to damaged microtubules. However, mitotic spindle abnormalities typically generate losses/gains of whole chromosomes (aneuploidy) whereas Ni(II) induced chromosomal gaps, translocations and fusions (41, 43). Consistent with the importance of genotoxicity, Ni(II)-transformed clones showed high levels of chromosomal damage (42, 78, 79). Our findings on the formation of DNA breaks by dNTP depletion and impaired repair of TOP1-DPC represent a more plausible, genotoxic mechanism for Ni(II)-induced formation of structural chromosomal abnormalities that are known to originate from DNA breaks and their inaccurate repair. Cells can tolerate only a limited number of gross chromosomal rearrangements, minimizing their contribution to the overall mutation burden in cells or at specific genes in the mutagenesis assays. Processing of DNA-embedded rNMPs by TOP1 is responsible for their mutagenicity which involves the generation of small 2 to 5 bp deletions in the short repeat sequences (17, 18, 19, 20). The nonrandom distribution of rNMPs-induced mutations is further enhanced by a strong preference (>20-fold) for AG and AC relative to AT dinucleotide deletions and exponentially higher deletion rates at di- and trinucleotide repeats with increasing tract length (80). Similar to Ni(II), established inhibitors of ribonucleotide reductase gallium and hydroxyurea were also nonmutagenic in the bacterial reversion assay and at the *Hprt* gene (81, 82), further demonstrating the inability of these standard tests to detect sequence-specific mutagenic activity of replication stressors promoting DNA-rNMPs incorporation and the subsequent formation of TOP1-DPC.

Ni(II) is a soft Lewis acid metal that binds to soft Lewis ligands such as Cys-SH and tertiary nitrogen in the imidazole ring of His (83). Ni(II) binding to a single ligand is relatively labile but coordination with two or more amino acids makes it much more stable. RRM1, the catalytic subunit of RNR, contains a large number of His and Cys residues (15 and 16, respectively), making it a multi-site target for Ni(II) binding with a potential for protein structure damage. Importantly, several Cys-SH are essential for RRM1 activity (84) and Ni(II) binding to any of these sites would completely inhibit critical redox reactions in RNR. The critical importance of several sterically accessible Cys-SH in RRM1 likely makes it also vulnerable to damage by many other endogenous and exogenous toxicants that display a strong reactivity toward SH-groups as evidenced by their ability to trigger activation of Cys-based cellular sensors of chemical stress (85). RRM1/RNR inhibition and the resulting increase in DNA-embedded rNMPs can lead to DNA breakage with carcinogenic consequences (22, 23) and cause neuroinflammation and neurodegeneration (6, 7, 8, 15). Our new assays for DNA-embedded rNMPs and TOP1-DPC offer sensitive and affordable methodologies for screening other seemingly nonmutagenic but protein-reactive carcinogens and toxicants for their potential ability to cause genomic instability and other pathophysiological changes *via* increased DNA incorporation of ribonucleotides and trapping of TOP1 as DPC. We speculate that mechanisms of downregulation of TOP1-DPC repair enzymes by Ni(II) are similar to those proposed above for RRM1. Mapping of specific amino acid residues bound by Ni(II) is needed to fully understand what makes some, but not many other proteins, targets for Ni-induced depletion/inactivation.

## Experimental procedures

### Chemicals

NiCl_2_ (N6136) was obtained from Sigma, AZD6738 from Cayman Chemical (21035), VE821 from Selleckchem (S8007), KU55933 from Selleckchem (S1092), KU60019 from Selleckchem (S1570), NU7026 from Calbiochem (260961), PD-0332991 from Selleckchem (S1116), RO3360 from Sigma Aldrich (SML0569), hydroxyurea from Sigma Aldrich (H8627), gemcitabine from Selleckchem (S1149).

### Cells and treatments

Human H460 and primary human WI38 and IMR90 cells were obtained from the American Type Culture Collection. WI38 and IMR90 cells were grown in HEPES- and glutamine-supplemented DMEM media (Gibco, 12430062) containing 20% fetal bovine serum. Primary cells were routinely maintained in a humidified atmosphere with 5% CO_2_ and 5% O_2_. H460 cells were grown under 5% CO_2_/20% O_2_ in 10% fetal bovine serum-containing RPMI-1640 (Gibco, 11875119). Cells were treated with a freshly prepared stock of NiCl_2_ in dH_2_O. Cells were synchronized in G1 and G2 phases by growth in the presence of 0.5 μM PD-0332991 and 10 μM RO3360 (16 h for H460 and 24 h for primary cells), respectively. In the indicated experiments, cell culture media was supplemented with 1 mM deoxyribonucleosides.

### Immunoblotting

Cells were treated at approximately 60 to 70% confluence and whole cell lysates were prepared by boiling for 10 min in a 2% SDS solution (2% SDS, 50 mM Tris-HCl pH 6.8, 10% glycerol, 5 mM EDTA). For immunoblotting of small proteins, samples were run on 12% SDS-PAGE gels and transferred onto PVDF membranes using PierceG2 Fast Blotter and 12% ethanol-supplemented buffer. Larger proteins were separated on 6% or 10% gels and then wet-transferred onto PVDF membranes at 4 °C overnight using Bjerrum Schafer-Nielsen Buffer. Primary antibodies: p53 (Santa Cruz, sc-126), phospho-S15-p53 (Cell Signaling, 9284), acetyl-K382-p53 (Cell Signaling, 2525), phospho-T21-RPA32 (Abcam, ab109394), phospho-S4/8-RPA32 (Bethyl, A300-245A), RPA32 (Millipore, MABE285), phospho-S139-H2AX (Cell Signaling, 9718), phospho-S317-CHK1 (Cell Signaling, 2344), CHK1 (Cell Signaling, 2360), phospho-T68-CHK2 (Cell Signaling, 2344), CHK2 (Cell Signaling, 3440), phospho-S824-KAP1 (Bethyl, A300-767A), KAP1 (Bethyl, A300-274A), phospho-S1981-ATM (Cell Signaling, 13050), ATM (Cell Signaling, 2873), phospho-T172-AMPK (Cell Signaling, 2531), phospho-S10-histone H3 (Cell Signaling, 9701), cyclin A (Sigma, c4710), cyclin E (Santa Cruz, sc-247), c-MYC (Cell Signaling, 13987), TYMS (Cell Signaling, 9045), RRM1 #1 Ab (Cell Signaling, 3388 - antibody used in all main Figures), RRM1 #2 Ab (Cell Signaling, 8837 - antibody used in validation and confirmatory studies in Fig. S3), RRM2 #1 Ab (Cell Signaling, 65939 - antibody used in all main Figures), RRM2 #2 Ab (Abcam, ab172476 - antibody used in validation and confirmatory studies in Fig. S3), RRM2B (Invitrogen, 79938), RNaseH2A (ThermoFisher, PA5-78330), RNaseH2B (ThermoFisher, PA5-83560), RNaseH2C (ThermoFisher, PA5-100321), HIF1α (BD Biosciences, 610958), HIF2α (Cell Signaling, 7096), MCL1 (Cell Signaling, 5453), MDM2 (Cell Signaling, 86934), RAD51 (GeneTex, GTX100469), PCNA (Santa Cruz, sc-56), L7A (Cell Signaling, 2415), γ-tubulin (Sigma, T6557), fibrillarin (Cell Signaling, 2639), GAPDH (Cell Signaling, 3683), lamin B1 (Cell Signaling, 12586), histone H3 (Cell Signaling, 9715), SUMO2/3 (Cell Signaling, 4971), topoisomerase I (GenTex, GTX55820), SPRTN (MyBioSource, MBS9131617), FEN1 (Cell Signaling, 83104), TDP1 (Cell Signaling, 59710), MUS81 (Santa Cruz, sc-53382), APEX2 (Cell Signaling, 74728), K48-linked polyubiquitin (Cell Signaling, 4289), PIAS4 (Cell Signaling, 4392), PIAS3 (Cell Signaling, 9042), RFN4 (R&D Systems, AF7964), UBC9 (Cell Signaling, 4786) and SOD1 (Cell Signaling, 2770). Primary antibodies were typically used at 1:1000 dilution. Horseradish peroxidase-conjugated goat anti-mouse IgG (Millipore, 12-349) and goat anti-rabbit IgG (Cell Signaling, 7074) were used as secondary antibodies and were added at 1:2000 dilution.

### Chromatin-bound proteins

H460 cells were collected by trypsinization, washed with cold PBS, and then incubated on ice for 20 min in 100 μl of 0.3% Triton X-100 buffer [0.3% Triton, 100 mM NaCl, 20 mM HEPES, pH 7.0, Halt Protease and Phosphatase Inhibitors (ThermoFisher Scientific)]. Soluble proteins were removed by centrifugation at 800*g* for 5 min at 4 °C. Pellets were resuspended in 50 μl of 0.3% Triton X-100 buffer, incubated for 5 min on ice, and centrifuged again. The insoluble cellular material was digested at 4 °C for 60 min with 100 units/ml benzonase in 100 μl of the lysis buffer supplemented with 2 mM Mg^2+^. Nuclease digestion was stopped by the addition of 5 mM EDTA and samples were centrifuged at 10,000*g* for 5 min at 4 °C. The supernatants were collected and used as a chromatin fraction.

### siRNA

Knockdowns of RNase H2 were generated using the ON-TARGETplus SMARTpool human siRNA from Dharmacon (RNaseH2A: L-003535-01-0020, RNaseH2B: L-014369-01-0020, RRM2: L-010379-00-0010). Controls were transfected with the ON-TARGETplus Control Pool Non-Targeting pool siRNA (Dharmacon, D-001810-10-10). Mixtures of siRNA (50 nM final concentration in media) and Lipofectamine RNAiMAX (Invitrogen, 13778150) were added to cells in 100-mm dishes for 6 h. The next day, cells were transfected again and seeded for the experiments 24 h later.

### Stable shRNA expression

H460 cells with a stable knockdown of p53 were generated using a continuous expression of shRNA from the chromosomally integrated pSUPER.retro.puro vector (OligoEngine,VEC-pRT-0002). The targeting sequence was 5′-GACTCCAGTGGTAATCTAC-3'. A control plasmid expressing a scrambled shRNA was obtained from the supplier of the vector. Annealed double-stranded oligonucleotides were ligated into the linearized vector DNA overnight using T4 ligase and the ligated products were transfected into *E. coli*. Purified pSUPER vectors were packaged into the viral particles in 293T cells that were cotransfected with MoMuLV gag-pol and VSVG-encoding plasmids. Virus-containing media was collected 48 h after transfections, filtered, and added to H460 cells overnight. Cells with stably integrated vectors were selected in the presence of 1.5 μg/ml puromycin.

### Immunofluorescence

H460 cells expressing nonspecific and p53-targeting shRNA were seeded at approximately 50% confluence on human fibronectin-coated coverslips (Corning, 356088). Next day, cells were treated with Ni(II) for 6 h. Replicating cells were labeled by the addition of 10 μM 5-ethynil-2′-deoxyuridine (EdU) for the last 1 h of Ni(II) treatments. Cells were fixed with 3.7% paraformaldehyde in PBS for 15 min at room temperature followed by permeabilization in PBS/0.5% Triton X-100 for 15 min at room temperature. Nonspecific binding was blocked with 2% fetal bovine serum in PBS at 37 °C for 30 min. DNA-incorporated EdU was labeled with Click-iT EdU-Alexa Fluor 488 Imaging kit (Invitrogen, C10337). Cells were incubated for 2 h at 37 °C with antibodies for phospho-Ser15-p53 (Cell Signaling, 9284) diluted with PBS containing 1% BSA and 0.5% Tween-20. Incubations with secondary Alexa Fluor 568 goat anti-rabbit antibodies (Life Technologies, A11036) were done for 1 h at room temperature in the dark. Coverslips were mounted on glass slides with the Vectashield DAPI mounting media (H-1200). Cells were viewed on the Nikon E−800 Eclipse fluorescent microscope (200–400× magnification, wide-field images). At least 100 cells in five or more randomly selected fields were scored per each slide.

### Cell cycle analysis

For accurate quantitation of S-phase populations, replicating cells were labeled with 10 μM EdU during the last hour of the drug treatments. Following collection by trypsinization, cells were fixed overnight in 80% ethanol at 4 °C, washed with PBS, and permeabilized with 0.5% Triton X-100 in PBS for 30 min at room temperature. After a wash with PBS, cells were incubated for 30 min at room temperature in the Click-iT reaction mixture (Click-iT EdU-Alexa Fluor 488 Flow Cytometry Assay kit from Invitrogen, C10420). Following a wash with PBS, cellular DNA was stained for 30 min with 0.5 μg/ml 7-AAD (BD Biosciences, 559925). Free 7-AAD was removed by a wash with PBS and cells were resuspended in 0.5 ml PBS for flow cytometry (FACSCalibur, BD Biosciences). Data were analyzed by the CellQuest Pro software.

### Gene expression

Total cellular RNA was extracted with the TRIzol Reagent (Ambion). The purity and concentrations of RNA preparations were determined by a Nanodrop spectrophotometer. Samples were stored at −80 °C. RNA was reverse-transcribed (RT First Strand Kit, Qiagen) and PCR reactions were set up using the RT SYBR Green ROX qPCR Mastermix and gene-specific primers were from Qiagen. Real-time PCR was performed using the ViiA7 Real-Time PCR System (Applied Biosystems). The expression of target genes was determined by the 2^−DDCt^ method. Three housekeeping genes (B2M, GAPDH, and TBP) were used for normalization purposes.

### Cellular Ni

Cellular uptake of Ni was measured as described previously (86) using nitric acid extracts of cells and graphite furnace atomic absorption spectroscopy (AAnalyst600 Atomic Absorption Spectrometer, PerkinElmer). This procedure was shown to recover >95% cellular Ni. Ni levels were normalized per cellular protein content in each sample or calculated as cellular concentrations using measurements of cellular volumes determined from forward scatter in flow cytometry (FACSCalibur, BD Biosciences).

### Measurements of cellular dNTPs

The amounts of cellular dNTPs were assayed using a modified enzymatic (primer extension) procedure which measures DNA synthesis supported by cellular extracts as a source of dNTPs (87). Two main modifications in our procedure were the use of fluorescent CF680R-dUTP (Biotium, 40003) instead of radioactive dNTPs and M13 ssDNA as a template for primer extension instead of synthetic oligos. The use of a larger template DNA allowed a simple and effective removal of unincorporated dUTP (and its fluorescent impurities) by Bio-Spin P30 columns (Bio-Rad, 7326231). H460 cells treated with a solvent, hydroxyurea (5 mM, 3 h) or Ni(II) (600 μM, 6 h) were collected by trypsinization, counted and 1.5 million cells were used for dNTP extraction. Cell pellets were resuspended in 200 μl of ice-cold 60% methanol and vortexed. Samples were incubated at 95 °C for 3 min and cooled to room temperature on lab bench. Supernatants were collected after centrifugation at 12,000*g* for 10 min at 4 °C. After drying of extracts in a Vacufuge at 60 °C, dNTPs were dissolved in 100 μl dH_2_O and stored at −80 °C. A260 measurements using a Nanodrop spectrophotometer showed <5% differences among extracts in different groups. A primer-template annealing mixture (vol = 50 μl, 10 μg M13 ssDNA, 40 pmol primer, 50 mM Tris-HCl, pH 8.0, 100 mM NaCl, 5 mM MgCl_2_) was heated at 95 °C for 5 min and then cooled down at 6 °C/min to 60 °C, held at 60 °C for 10 min and then cooled to room temperature at 6 °C/min. Primer DNA (CCGCTCACAATTCCACACAAC) was obtained from Integrated DNA Technologies (custom single-stranded DNA oligo), M13 ssDNA (N4040S), and Taq polymerase (M0267L) were from New England Biolabs. Primer extension reactions (vol = 20 μl) contained 0.4 μg M13 DNA (from the primer-annealed mix), 5 μM CF680R-dUTP, 2 units Taq polymerase, Taq polymerase buffer and 10 μl cellular extracts or dNTP mix. Samples were incubated for 60 min at 48 °C and then passed through Bio-Spin P30 columns equilibrated in the buffer containing 100 mM NaCl, 10 mM Tris, pH 8.0 and 10 mM EDTA. DNA samples (0.3 μg) were run on 1% agarose-TBE followed by scanning of gels using a Li-Cor Odyssey CLx (685 nm excitation laser/700 nm emission channel). Next, DNA was stained for 30 min with GelRed (Millipore, SCT123) and imaged with a BioRad Gel Doc XR+. DNA-incorporated dUMP fluorescence was normalized per amount of DNA. The value of dUMP signal/DNA in blank reactions (CF680R-dUTP only without other dNTPs) was used for background subtraction. The inclusion of the high concentration (5 μM) of CF680R-dUTP ensured that DNA labeling was insensitive to fluctuations in the sample dTTP levels. The overall DNA synthesis was proportional to the levels of three remaining dNTPs.

### FL-RIB (Fluorescent Labeling of RNaseHII-Induced breaks) assay for DNA-embedded rNMPs

DNA was isolated using the PureLink Genomic DNA Mini Kit (Invitrogen, K182001) with some modifications. Cells were collected with trypsin, resuspended in 200 μl of Buffer A for RNA digestion (10 mM HEPES, pH 7.0, 100 mM NaCl, 1% NP40, and 400 μg RNase A from the kit) and incubated for 20 min at 4 °C. Soluble material was removed by centrifugation at 800*g* for 5 min at 4 °C. Nuclear pellets were then resuspended in 200 μl PBS and the subsequent steps in DNA purification followed the kit protocol. DNA was eluted from columns with water and its concentration was measured using a Nanodrop spectrophotometer. In some experiments (hydroxyurea-treated cells), we also successfully used DNA isolated by a kit from Qiagen (13323). Tubes were put on ice to assemble nick-labeling reactions (vol = 20 μl) which contained 2 μl 10xNEBuffer 2 (New England Biolabs, B7002S), 0.1 to 0.5 μg DNA, and 1 μl RNase HII (5000 units/ml, New England Biolabs, M0288S) or H_2_O for mock digestions. Samples were incubated for 2 h at 37 °C. Tubes were placed on ice and 2 μl dNTP mix and 0.5 μl DNA polymerase I (10 units/μl, New England Biolabs, M0209S) were added. The dNTP mix contained 200 μM dATP, dCTP, dGTP, 100 μM dTTP (New England Biolabs, N0446S), 100 μM dye-labeled dUTP (Atto680-dUTP from Jena Bioscience, NU-803-680-L or CF680R-dUTP from Biotium, 40003) and 20 mM Tris-HCl, pH 8.0. Samples were then incubated for 1 h at 16 °C. The nick-labeling reactions were stopped with the addition of 5 μl of 0.5 M EDTA, pH 8.0, and placement of the tubes on ice. For Atto680-dUTP batches with low amounts of impurities, samples were mixed with a loading dye solution (New England Biolabs, B7021S) and run on 1% agarose-TBE gels to separate labeled DNA from a free Atto680-dUTP. For Atto680-dUTP batches with higher levels of impurities that moved only slightly slower than labeled DNA and for all CF680R-dUTP reactions, samples were passed through Bio-Spin P30 gel columns (Bio-Rad, 7326231) to remove free dUTP-dye conjugates and their impurities. P30 columns were equilibrated in the buffer containing 100 mM NaCl, 10 mM Tris, pH 8.0, and 10 mM EDTA. The gels were first imaged with a Li-Cor Odyssey CLx (685 nm excitation laser/700 nm emission channel for both dyes), then DNA was stained for 30 min with GelRed (Millipore, SCT123) and imaged with a BioRad Gel Doc XR+. dUTP incorporation (fluorescence) was normalized per amount of DNA and the ratios of dUTP signal/DNA in mock digestions was subtracted from those in RNase HII-digested samples to obtain rNMP-specific values.

### Detection of DNA-protein crosslinks (DPC) and their modifications: DPC-trap assay

Cells were seeded at approximately 60% confluency into 60 mm dishes. After treatment, the media was removed, cells were washed twice with cold PBS and collected by scraping in 2% SDS lysis buffer (2% SDS, 20 mM Tris-HCl pH 8.0, 5 mM EDTA, 50 mM DTT from a freshly prepared stock, freshly added 1 mM PMSF). Samples were boiled for 5 min to inactivate ubiquitin and SUMO proteases and prevent reversal of Top1-DPC. After cooling down, DNA was sheared 5 times with a 25 Ga needle. NaCl was added to a final concentration of 200 mM followed by two volumes of ethanol. Tubes were inverted several times and left at 4 °C overnight. Next day, samples were spun at 10,000*g* for 5 min at room temperature. Supernatants were discarded and pellets were washed twice with 1 ml of 70% ethanol (10,000*g*, 5 min). Pellets were air-dried and dissolved in 0.1 ml of 1% SDS by heating for 20 min at 50 °C. Dissolved DPC/cell precipitates were stored at −80 °C. DPC samples were mixed with the gel loading buffer, boiled for 10 min, and loaded onto 6% gels with 4% stacking gel. Following electrophoresis, the gels were washed two times with 20 mM HEPES, pH 7.4 for 5 min at room temperature. With the gels covered in 20 mM HEPES pH 7.4, 5 μM bleomycin and 5 μM Fe-citrate (from separate stocks) were added and gels were incubated for 5 min at room temperature. DNA cleavage was activated by the addition of 1 mM ascorbate and incubation for 20 min at room temperature. The chemical nuclease-containing solution was removed and gels were rinsed with the SDS-PAGE running buffer containing 0.1 mM EDTA. Gels were washed two times with the same solution for 5 min each. The gels were then used for standard wet transfer of proteins onto PVDF membranes followed by immunoblotting for Top1, SUMO2/3, or K48-linked polyubiquitin.

### Cytotoxicity

Cell viability was measured using the CellTiter-Glo luminescent assay (Promega, G7571). Cells were seeded (500–1000 per well) into optical bottom cell culture plates (ThermoFisher Scientific, 165305) and grown overnight before treatments. Assay measurements were taken at different times after the removal of chemicals.

### Colony formation assay

H460 cells were seeded onto 6-well plates (200 cells/well) and allowed to attach overnight. The medium was replaced and cells were continuously treated with Ni(II) until visible colonies were formed. Media was refreshed once after 4 days of treatments with Ni(II). Colonies were fixed with methanol and stained with a Giemsa solution.

### Statistics

Statistical significance between two independent groups was evaluated by two-tailed, unpaired *t* test. Data in multiple comparisons were evaluated by one-way ANOVA with the post-hoc Tukey's test. Differences with *p* < 0.05 were considered statistically significant.

## Data availability

All data supporting the findings of this study are available within the article and Supplementary Information. The authors state that all data necessary for confirming the conclusions presented in the article are represented fully within the article.

## Supporting information

This article contains supporting information.

## Conflict of interest

The authors declare that they have no known competing financial interests or personal relationships that could have appeared to influence the work reported in this paper.
