# Supplementary material for: Atypical genotoxicity of carcinogenic nickel(II): Linkage to dNTP biosynthesis, DNA-incorporated rNMPs, and impaired repair of TOP1-DNA crosslinks
Source: J Biol Chem. 2023 Oct 25;299(12):105385. doi: 10.1016/j.jbc.2023.105385 (PMC10692736; doi:10.1016/j.jbc.2023.105385)
Supplement: Supporting Information [file mmc1.docx]

**Atypical genotoxicity of carcinogenic nickel(II): linkage to dNTP biosynthesis, DNA-incorporated rNMPs and impaired repair of TOP1-DNA crosslinks**

Casey Krawic, Michal W. Luczak, Sophia Valiente and Anatoly Zhitkovich

List of materials included in Supporting Information:

Figure S1

Figure S2

Figure S3

**
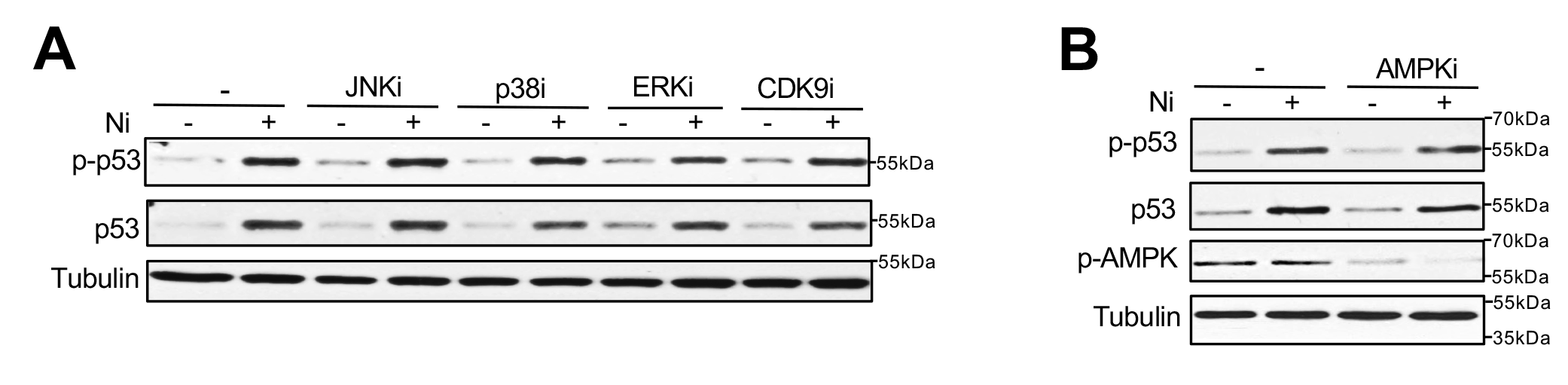
**

**Figure S1. AMPK, CDK9 and cytoplasmic SAPKs (JNK, p38 and ERK) are dispensable for p53 activation by Ni(II).** H460 cells were cotreated with Ni(II) for 6 h and various inhibitors. AMPKi: 1 μM compound C, JNKi: SP600125, p38i: SB203580, ERKi: FR180204, CDK9i: CDK9 inhibitor II from Santa Cruz (all at 10 μM). (**A**) S15-p53 phosphorylation and p53 protein levels in the presence of inhibitors of CDK9 and cytoplasmic stress-activated protein kinases (SAPKs). (**B**) Activation of p53 and AMPK-T172 phosphorylation in AMPK-inhibited cells.

**
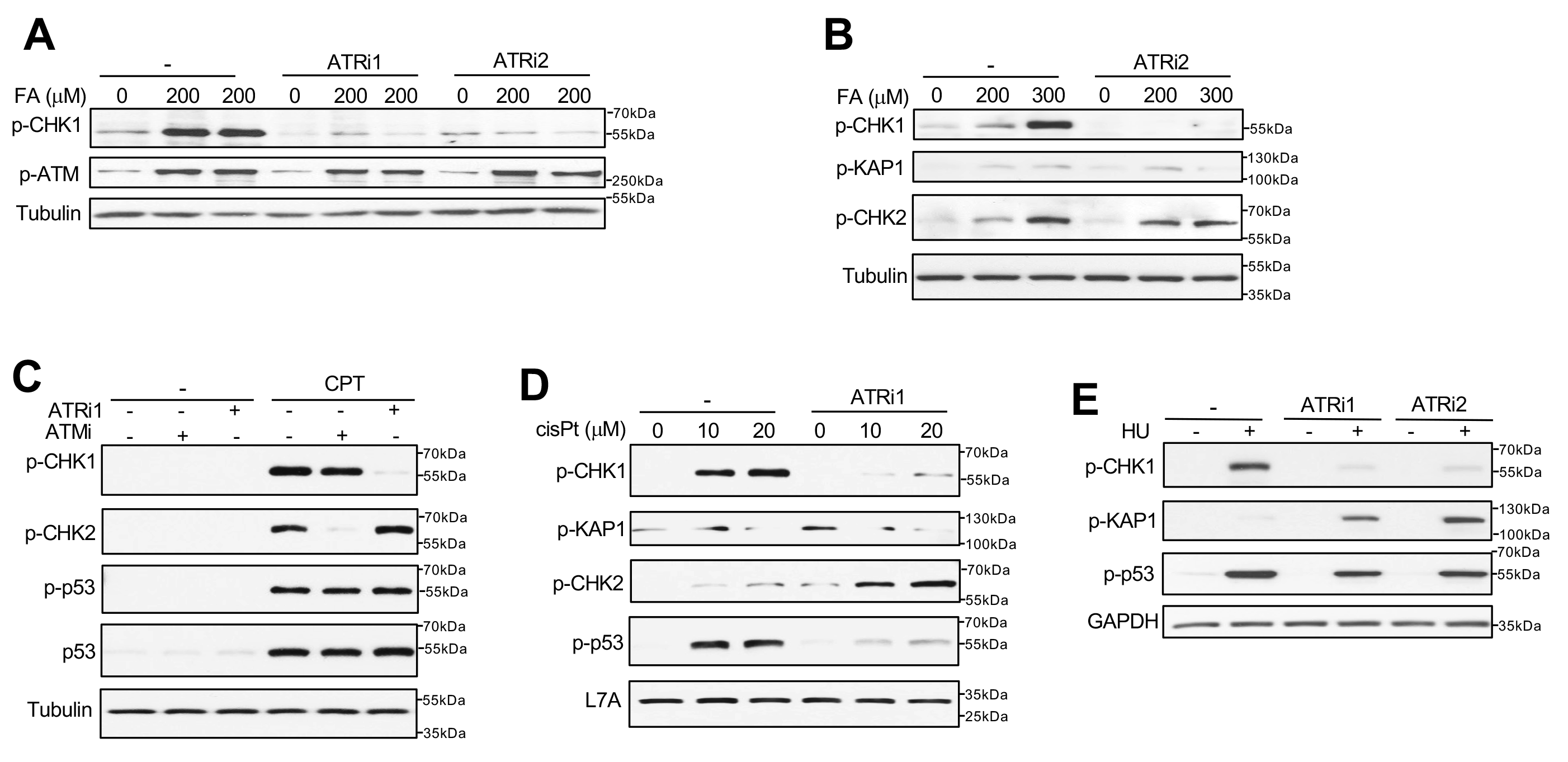
**

**Figure S2. Impact of ATR inhibition on ATM activation by known replication stressors.** Cells were preincubated with ATR inhibitors (ATRi1: 0.5 μM AZD6738, ATRi2: 10 μM VE821) for 30 min prior to treatments for 3 h with replication stressors: formaldehyde (FA), camptothecin (CPT), cisplatin (cisPt) or hydroxyurea (HU). (**A**) CHK1 phosphorylation and ATM activation readouts in H460 and (**B**) IMR90 cells treated with FA in the presence of ATR inhibitors. (**C**) DNA damage responses in cells treated with CPT in the presence of ATR and ATM inhibitors (ATMi: 10 μM KU55933). (**D**) Phosphorylation of ATM and ATR targets in H460 cells treated with cisPt alone and in the presence of ATRi1. (**E**) CHK1, KAP1 and p53 phosphorylation induced by 5 mM HU in H460 cells in the presence of ATR inhibitors.

**
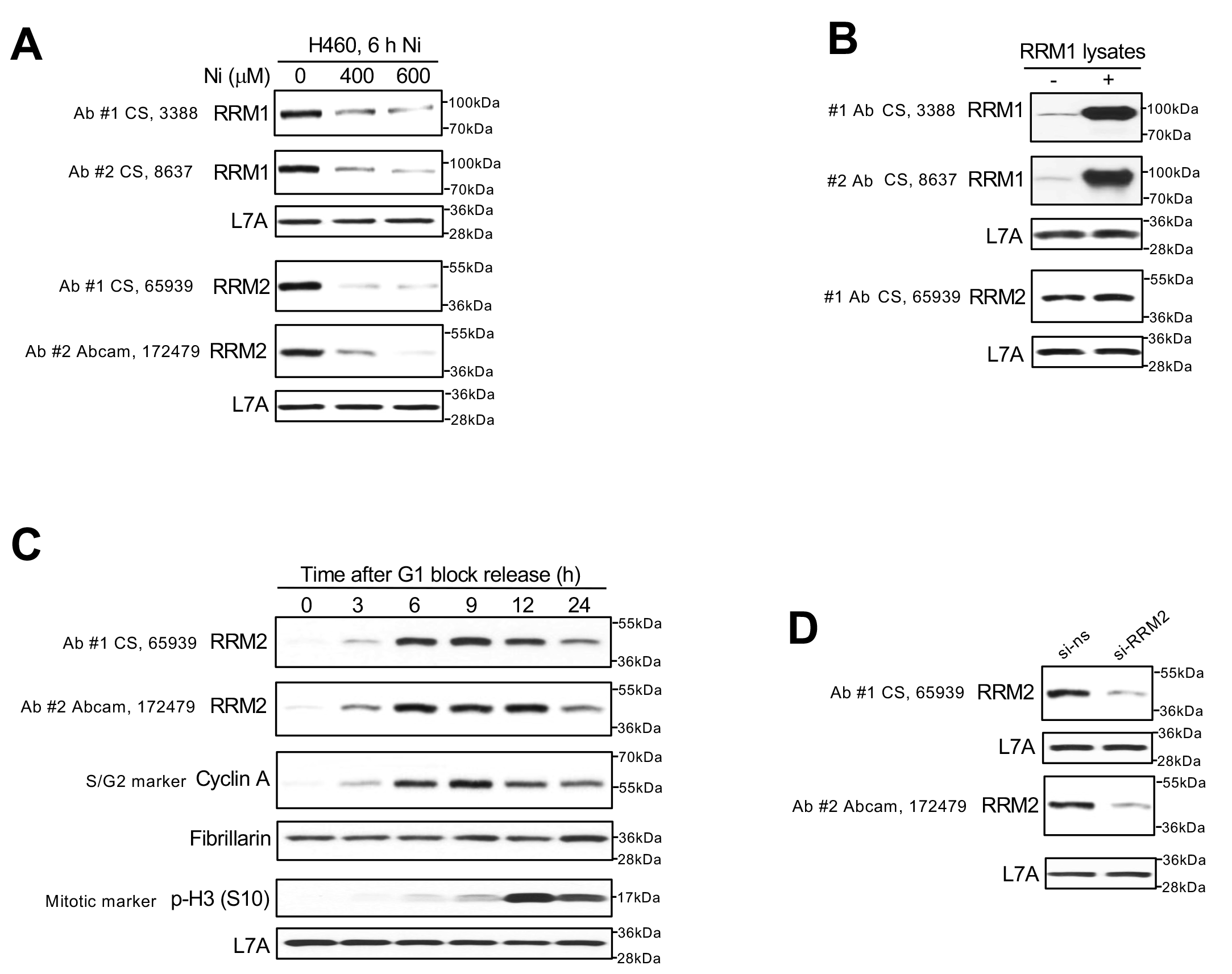
**

**Figure S3. Validation of anti-RRM1 and anti-RRM2 antibodies. (A)** Similar changes in RRM1 and RRM2 abundance detected by two antibodies for each protein. H460 cells were treated with Ni(II) for 6 h. **(B)** Both RRM1 antibodies, but not RRM2 antibody, detected spiking of H460 samples with RRM1-overexpressing lysates. Loaded samples included 20 μg H460 lysates mixed with either 3 μg control or RRM1-overexpressing lysates (Origene, LY422336). **(C)** Physiological validation of RRM2 antibodies via analysis of RRM2 expression during cell cycle. H460 cells were arrested in G1 phase by 0.5 μM PD-0332991 for 16 h and then released from G1 block by growth in a drug-free media and collected at different times. **(D)** Decreased expression of RRM2 detected with two antibodies in WI38 cells transfected with RRM2-trageting siRNA.
